# Triggering of viral and bacterial respiratory infection hospitalizations by traffic pollution exposure in a cohort of hospitalized adults

**DOI:** 10.1371/journal.pone.0352323

**Published:** 2026-07-13

**Authors:** Daniel P. Croft, Md Rayhanul Islam, Kelly Thevenet-Morrison, Carl J. Johnston, Mark J. Utell, Philip K. Hopke, Steve N. Georas, Augusto A. Litonjua, Derick R. Peterson, Soumyaroop Bhattacharya, Andrea Baran, Chinyi Chu, Anthony Corbett, Michael Peasley, Angela R. Branche, Edward E. Walsh, Matthew N. McCall, Sally W. Thurston, Ann R. Falsey, Thomas J. Mariani, David Q. Rich

**Affiliations:** 1 Department of Medicine, Pulmonary and Critical Care Medicine Division, University of Rochester Medical Center, Rochester, New York, United States of America; 2 Department of Environmental Medicine and Public Health Sciences, University of Rochester Medical Center, Rochester, New York, United States of America; 3 Institute for a Sustainable Environment, and Center for Air Resources Engineering and Science, Clarkson University, Potsdam, New York, United States of America; 4 Department of Pediatrics, University of Rochester Medical Center, Rochester, New York, United States of America; 5 Department of Biostatistics and Computational Biology, University of Rochester Medical Center, Rochester, New York, United States of America; 6 Clinical and Translational Science Institute, University of Rochester Medical Center, Rochester, New York, United States of America; 7 Department of Medicine, Infectious Diseases Division, University of Rochester Medical Center, Rochester, New York, United States of America; 8 Department of Biomedical Genetics, University of Rochester Medical Center, Rochester, New York, United States of America; The University of Tokyo Graduate School of Medicine Faculty of Medicine: Tokyo Daigaku Daigakuin Igakukei Kenkyuka Igakubu, JAPAN

## Abstract

The rate of respiratory viral infection (RVI) associated with acute air pollution exposure is well established, but whether bacterial and viral infections respond similarly to traffic related air pollution (TRAP) exposure is less well understood. Using a novel seasonal time-stratified case-crossover design and conditional logistic regression, we separately estimated the rate of hospitalization for 465 patients with RVI, respiratory bacterial infection (RBI), or combined respiratory viral and bacterial infection (RVBI) associated with increased ambient particulate matter ≤2.5 µm (PM_2.5_), black carbon (BC), nitrogen dioxide (NO_2_) and carbon monoxide (CO) concentrations in the previous 1, 2, and 3 weeks (lag days 0–6, 7–13, 14–20). In a novel approach, a four-physician panel adjudicated each case of infection to accurately classify the type of infection present and only patients with the highest diagnostic certainty were enrolled in this study. Associations were strongest between TRAP and RVI at the 0–6 lag period, with fewer, less precise associations at later lag times for RVBI and RBI. Each 2.6 µg/m^3^ increase in PM_2.5_ on lag days 0–6 was associated with a 22.1% increased rate of RVI hospitalization (95% CI: 1.6%, 46.7%). Each 0.1 µg/m^3^ increase in BC was associated with a 30.0% increase (95% CI: 5.0%, 61.1%) in the rate of hospitalization for RVI. Rates of hospitalization for RVI associated with increased PM_2.5_ were generally largest for lag days 0–6 and 7–13. The RVI/BC rate ratio was larger for females than males for days 0–13, but not for PM_2.5_ and NO_2_. Short term increases in PM_2.5_, BC, NO_2_, and CO concentrations (markers of traffic pollution) were associated with an increased rate of RVI hospitalization, while sex-specific associations were observed between BC and RVI for females. Further study of the mechanism underlying the effect of TRAP on RVI is needed.

## Introduction

Outdoor (ambient) air pollution has been established globally as a risk factor for respiratory viral infection (RVI) in adults in both epidemiology studies and experimental animal models [1–3]. This association has been observed in both high and low air pollution settings, including Rochester, New York where annual average PM_2.5_ concentrations are below the national ambient air quality standard.

Two prior New York State studies have observed increased rates of hospitalization for RVI associated with increased ambient PM_2.5_ concentrations in the previous few days and previous week, but not increased rates of respiratory bacterial infection (RBI) [4,5]. We have also reported associations between the rate of RVIs and specific constituents of PM_2.5_ in New York State (spark-ignition emissions, diesel, biomass burning, road dust, pyrolyzed organic rich, secondary nitrate and secondary sulfate) [6,7] and others have reported associations between RVI and pollutants considered to be markers of traffic pollution (e.g., nitrogen dioxide [NO_2_], carbon monoxide [CO], and black carbon [BC]) [2,8–10]. Given the likelihood for outcome misclassification in these prior studies using large administrative databases, it is important to estimate and compare the specific associations between a broad array of pollutants and rigorously confirmed diagnoses of RVI, RBI and combined respiratory viral and bacterial infection (RVBI).

Multiple patient and community factors may modify an individual’s risk of respiratory infection associated with air pollution exposure. The association between air pollution and RVI may be sex-specific since males may experience worse respiratory tract inflammation when exposed to air pollution prior to an infection, than females [11]. Second, combustible cigarette smoking is a direct, complex gaseous and particulate pollutant exposure [12], which is an established risk factor for respiratory infections [13], and can modify the association between air pollution and respiratory infection [14]. Capturing this intense exposure (which has significant overlap in composition with ambient air pollution) is important within population health studies as direct cigarette exposure is often not accurately assessed in ambient air pollution estimates. Third, air pollution has been described a risk factor for respiratory viral infection in patients with COPD [15] and asthma [16] but the relationship between air pollution and respiratory infection in patients with obstructive lung diseases (COPD and asthma) remains an active area of study. Lastly, historic redlining produced segregated urban neighborhoods by race leading to environmental justice concerns and health disparities, including poor asthma outcomes in urban areas of Pittsburgh and California [17,18]. Exploration of how these features of poverty and environmental justice concerns affect a community’s RVI response to communal ambient air pollution exposure is thus needed.

Within a cohort study of rigorously adjudicated RVI, RBI and RVBI diagnoses in adults living in Rochester, New York, we hypothesized that increased weekly traffic air pollutant concentrations would be associated with increased rates of respiratory infection hospitalization, with larger rate ratios for RVI than either RBI or RVBI. Further, based on our prior research, we expected the rate of RVI hospitalization associated with increased pollutant exposure to be higher in the week prior to viral infection acquisition (e.g., lag days 7–13), than during infection (e.g., lag days 0–6). We also explored stratification of these associations by sex, smoking status, distance from the central monitor and neighborhood deprivation.

## Methods

### Study population and protocol

N = 1103 patients completed informed written consent and were enrolled into a NIH funded prospective cohort study focused on development of a gene classifier to discriminate between bacterial and non-bacterial respiratory illness (Protocol #1889) at the University of Rochester Medical Center (URMC) [19]. At enrollment into protocol #1889, patients provided written consent for secondary analyses. This study is a planned secondary analysis estimating the rate of respiratory infection associated with increased TRAP concentrations in the 1–3 weeks prior to hospitalization for RVI, RBI or RVBI. Research studies review board (RSRB) approval was obtained for this study (Protocol #5233) at URMC. Patients were enrolled within 24 hours of admission to the two largest medical centers (Strong Memorial Hospital and Rochester General Hospital) in Rochester, New York between March 1, 2019 and April 30, 2023. However, enrollment was paused from March 2020 to October 2020 due to the COVID-19 pandemic. Inclusion criteria included age >18 years and respiratory symptoms consistent with an acute respiratory infection. For our analyses of the cohort study, participants were then excluded if they had an immunosuppressive condition, were taking immunosuppressive medications, were infected with COVID-19 (due to the complexity of pandemic dynamics), were on antibiotics greater than 24 hours prior to enrollment, or were unable to provide consent. Though COVID-19 was excluded, all other types of RVI were included (S1 Table in S1 File). Only the first hospitalization was included for patients who were admitted multiple times, leaving 465 patients for the analyses described below (S1 Fig in S1 File). Smoking status, presence of comorbidities like COPD and asthma, and home location were all verified with participants.

### Respiratory infections

Upon enrollment, demographic and clinical information (e.g., full medical history including laboratory and microbiologic data and timing of symptoms) were collected. Following data collection, a four-member panel (three infectious disease specialists and one pulmonary and critical care medicine specialist) independently adjudicated each participant’s index respiratory infection as a RVI, RBI or a RVBI. There were no predetermined criteria dictating how to weigh the available clinical histories, hospital course, imaging, laboratory findings including serum biomarkers and microbiologic data. Each clinician used their own clinical experience and all available data (including PCR or sputum culture positivity, imaging, patient history and the electronic medical record) to make a determination as to the certainty of the microbiologic classification (i.e., Definite, Probable). Only definite RVI, RBI or RVBI infections were included. For example, a definite RVI was classified as a patient with respiratory symptoms consistent with infection, a positive viral PCR from a multiplex viral nasal swab, and an absence of evidence of a bacterial infection. In a similar manner, definite RBI required convincing evidence of a respiratory bacterial infection and absence of viral infection. RVBI required a high certainty of both RVI and RBI. After individual assessments were made, all four members of the adjudication team convened for adjudication meetings to discuss any discordance in assessment of type of infection (RVI, RBI or RVBI) or clinical scenario (pneumonia or non-pneumonia). The concordance was highest for RVI (98.5%), next highest for RBI (93.8%) and lowest for RVBI (90.3%). A final decision was made during this adjudication meeting. Of note there were uncommon cases when only three members reviewed each participant’s data, but at the very least, three reviewers evaluated each patient and came to consensus on any discordance assessment. To our knowledge, no prior population epidemiology study of air pollution and respiratory infection has more rigorously verified RVI, RBI and RVBI than our current study.

### Ambient air pollution and meteorology data

PM_2.5_ was measured at the New York State Department of Environmental Conservation site (latitude 43°09′56″ N, longitude 77°33′15″ W) at the intersection of two major interstates (I-490 and I-590) using a Teledyne API T640 PM_2.5_ Particle Monitor. A 2-wavelength aethalometer was used to measure black carbon (BC) at 880 nm with Delta-C (DC) concentrations calculated as the difference between black carbon (BC) measured at 370 nanometers and that measured at 880 nanometers. DC provides a tracer for wood smoke and biomass burning [20]. Concentrations of ultrafine particles (UFP, < 100 nm; particles/cm^3^) and accumulation mode particles ([AMP] 100–500nm; particles/cm^3^) were measured with a scanning mobility particle sizer (SMPS, TSI, Inc, Shoreview, MN) [21]. Nitrogen dioxide (NO_2_), sulfur dioxide (SO_2_), carbon monoxide (CO) and ozone (O_3_) were measured as a part of the State and Local Air Monitoring Stations (SLAMS) using Federal Equivalent Method (FEM) gas monitors (ThermoFisher Scientific, Inc, USA). Although CO is not traditionally thought to have direct effects on respiratory infection outcomes, it serves as a marker of traffic pollution exposure [22]. Due to the use of central site estimates and the effect of localized sources, particle chemistry and aerosol dynamics, it is reasonable to anticipate greater heterogeneity in the NO_2_ and BC concentrations compared to the PM_2.5_ values across the study area [23]. These pollutants were included in the statistical analyses described below.

### Statistical analysis

**Fig 1:**
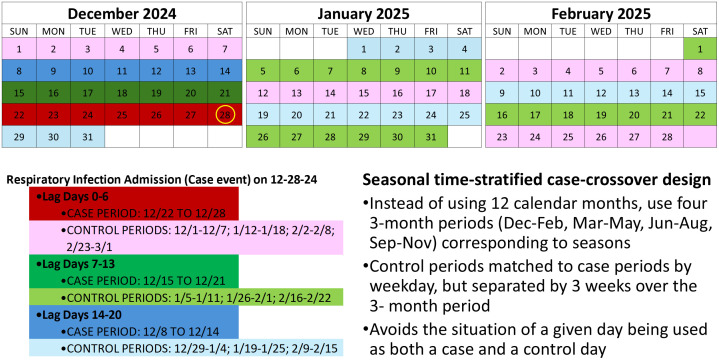
Example of case and control period selection in the seasonal time-stratified case-crossover design.

We used a novel modification of the standard time-stratified case-crossover design [24,25] and conditional logistic regression analyses to estimate the rate of respiratory infection associated with increased air pollutant concentrations in the previous 1, 2, and 3 weeks. The standard time-stratified case-crossover design defines a 1-week case period as the day of hospitalization and the previous 6 days (e.g., December 22–28) and control periods as all the 1-week periods before and after this period within December (December 1–7, 8–14, and 15–21, and December 29-January 4). Air pollutant concentrations for these case and control periods are then contrasted. However, use of this standard time-stratified case-crossover study design to estimate the rate of respiratory infection associated with increased pollutant concentrations in multiple lag weeks (i.e., previous 1 week [lag days 0–6], 2 weeks [lag days 7–13], and 3 weeks [lag days 14–20]) is problematic because the air pollutant concentration on some calendar days are included as a control period concentration in one weekly analysis (e.g., lag days 0–6) and then as a case period concentration in an adjacent weekly analysis (e.g., lag days 7–13). If we observed an increased rate of respiratory infection associated with an increased pollutant concentration in lag days 0–6, we would likely observe a decreased rate associated with increased pollutant concentration in lag days 7–13. Therefore, we applied a “seasonal time-stratified” case-crossover design to avoid this problem, where season (Winter: December – February; Spring: March – May; Summer: June – August; Autumn: September – November) was used to define case and control periods for each individual patient, rather than calendar month as is done in the standard time-stratified case-crossover design [24]. For example, for each respiratory infection hospitalization (e.g., hospitalization on December 28), the day of the hospitalization and the 6 prior days were the case period (e.g., December 22–28), while control periods were defined as 1-week periods that are each 3 weeks before and after the case period and other control periods within the same season (e.g., December 1–7, January 12–18, February 2–8, and February 23-March 1) (Fig 1). Case periods for lag days 7–13 and 14–20 were defined in the same manner. This is the first use of the seasonal time stratified case-crossover design that allows for the inclusion of lag periods past 1 week, without days being included in both case and control periods for different lagged analyses.

These three lag periods (lag days 0–6, 7–13, 14–20) are the same as the lag periods used in previous studies (though prior studies did not use the novel seasonal time-stratified case-crossover design).The individual week lag periods are reasonable intervals due to the variability in the number of days between infection and the start of symptoms (incubation period) and the number of days of symptoms prior to presenting to the hospital. For RVI, the one week period before hospitalization (i.e., lag days 0–6) roughly corresponds to a period during infection, while lag days 7–13 correspond to early infection or time prior to infection. Lag days 14–20 are clearly prior to infection for RVI (S2 Fig in S1 File). Due to the complicated nature of RBI and the potential of RBI being preceded by RVI, the portions of RBI infection to which these lag times correspond (e.g., prior to, or during infection) is discussed in further detail in the supplement (and S3 Fig in S1 File). Though the novel seasonal modification is pragmatic, it still has limitations including 3 weeks being the maximal lag period possible within the seasonally modified design. Including a fourth week (days 21–28) would not be possible due to the potential for days being present in both case and control periods. To include a fourth week, the time period would need to be expanded to 4 months (which would extend past an individual season). Because case and control periods were from the same patient in the case-crossover study, time-invariant confounders such as age, gender, and comorbidities were the same for case and control periods and thus, controlled by design. Factors like temperature and relative humidity that varied between the case and control periods were potential confounders and had to be included in the analytic models described below.

Using conditional logistic regression, stratified by matched case/control set, we regressed case-control status (case = 1, control = 0) against the mean PM_2.5_ concentration in the previous 1 week (lag days 0–6). In this model, we also included the mean temperature and relative humidity during the same lag days. Using Akaike’s information criterion, we determined whether temperature and relative humidity should be modeled as a linear term (1 degree of freedom [df]) or a non-linear term (2, 3, or 4 df), with 1 df determined to be optimal for both mean temperature and relative humidity. From this model, we estimated the rate of RVI (and its 95% confidence interval [CI]) associated with each interquartile range increase in PM_2.5_ concentration in the previous week (lag days 0–6). We then re-ran this model for each combination of outcome (RVI, RBI, and RVBI), pollutant (NO_2_, CO, BC, DC, UFP, AMP, SO_2_, and O_3_), and lag time (lag days 0–6, 7–13, and 14–20). Though statistical significance in this study was defined as p < 0.017 (3 lag weeks examined), we made inference on the magnitude and direction of the effect estimates rather than on statistical significance. Making inference on magnitude and direction of effect estimates is particularly relevant in this study as the many statistical models conducted in this analysis increases the likelihood of observing statistically significant associations purely by chance.

### Sensitivity analyses

We conducted multiple exploratory stratification analyses. To determine if our findings were robust to the type of time-stratified design applied, we estimated the rate of RVI associated with air pollutant concentration on lag days 0–6 using both the modified seasonal time-stratified case-crossover design and the standard time-stratified design. To adjust for social and economic forces that could affect patients, we then conducted exploratory analyses including whether associations were modified by Area deprivation index (ADI), a measure of poverty in the neighborhood [26]. Area deprivation index uses factors related to income, education, employment and housing quality that allow for neighborhoods (census block group level) to be ranked by socioeconomic disadvantage. Using patients’ home addresses to place them into census block groups, we stratified patients into those with an ADI decile in New York State of <9 versus those with an ADI decile of 9 or 10 (higher deprivation) and then re-ran the main analyses described above, including an interaction term between pollutant and ADI (e.g., PM_2.5_*ADI) in the model. We also explored whether or not the distance from the central air quality monitor affected the rate of hospitalization, using two distances (less than or equal to 5 miles [median of population] or over 5 miles from the monitor [S6 Fig and S7 Table in S1 File]). Last, we stratified the study sample by sex, smoking status (active, former or never) and obstructive lung disease (presence of Asthma/COPD or not) and re-ran the main analyses described above in each strata of sex, smoking status and lung disease category.

## Results

Of the 465 adult patients hospitalized with respiratory infection with microbiologic classifications, 55% (n = 256) were diagnosed with RVI, 27% (n = 125) had RBI, and 18% (n = 84) had RVBI (Table 1). The median age was 63 years old. The majority of patients with RVIs were female (64%), while RBI patients were predominantly male (60%). Overall, study participants were mainly white (67%) ninety percent were community dwelling at the time of hospitalization, while the remaining participants were unhoused, in assisted living, or resided in nursing homes (10%). The most common comorbidity was hypertension (60%), followed by asthma (31%), COPD (26%), diabetes (32%), high cholesterol (24%), atherosclerotic heart disease (16%), and obesity (11%). Large proportions of patients with asthma and COPD had a viral infection (69% and 45% respectively). Patients with RVI reported an average of 4 days between the dates of symptom onset and presentation to the emergency department, while patients with RBI and RVBI reported symptom onset of 6 days prior to presentation. The most common viral infections were Influenza A (35%) and Rhinovirus (29%), while the most common bacterial infections were *Haemophilus influenzae* (11%) and Streptococcus infections (10%) (S1 Table in S1 File). Most patients (75%) were either actively smoking within the last three months or had a history of past smoking. Only 9% of the patients needed 24-hour oxygen support at baseline.

**Table 1:** Characteristics of study population, by infection type.

		Overall n (%) (n = 465)	Viral n (%) (n = 256)	Bacterial n (%) (n = 125)	Coinfection n (%) (n = 84)
**Characteristic**					
**Age (years), Median (IQR)**		63(22)	62(25)	65(18)	61(19)
**Gender**	Female	259(56)	162(63)	51(41)	46(55)
**Race**	White	311(67)	161(63)	93(74)	57(68)
	African American	141(30)	86(34)	30(24)	25(30)
	American Indian or Alaska Native	1(0)	1(0)		
	Asian	1(0)	1(0)		
	Native Hawaiian or Other Pacific Islander	1(0)	1(0)		
	Multi racial	5(1)	2(1)	2(2)	1(1)
	Unknown	5(1)	4(2)		1(1)
**Ethnicity**	Hispanic	52(11)	34(13)	10(8)	8(10)
**Living needs**	Independent	418(90)	228(89)	112(90)	78(93)
**Lung Disease Category**					
	COPD	102(25)	46(21)	32(29)	24(33)
	Asthma	124(31)	87(39)	16(14)	21(29)
	No lung disease	180(44)	88(40)	64(57)	28(38)
**Additional comorbidities**					
**Diabetes**		144(31)	80(31)	35(28)	29(35)
**Congestive Heart Failure**		55(12)	31(12)	17(14)	7(8)
**Atrial fibrillation**		51(11)	28(11)	15(12)	8(10)
**Hypertension**		279(60)	151(59)	79(63)	49(58)
**High Cholesterol**		112(24)	68(27)	20(16)	24(29)
**Atherosclerotic Heart disease**		76(16)	48(19)	16(13)	12(14)
**Obesity**		66(14)	46(18)	10(8)	10(12)
**Esophagitis/ acid reflux**		99(21)	57(22)	23(18)	19(23)
**Other Psychiatric**		42(9)	25(10)	8(6)	9(11)
**Depression**		91(20)	49(19)	19(15)	23(27)
**Sleep Apnea**		46(10)	25(10)	11(9)	10(12)
**BMI Median (IQR)**		29.2(11)	30.8(12.3)	29(9.9)	27.3(9.7)
**Tobacco smoke**	Never	119(26)	76(30)	27(22)	16(19)
	Active (within 3 months)	160(34)	81(32)	45(36)	34(41)
	Past	185(40)	99(39)	53(42)	33(40)
**Oral Steroid**	Yes	30(6)	19(7)	5(4)	6(7)
	No	434(94)	237(93)	119(96)	78(93)
**Inhaled Steroid**	Yes	169(37)	97(38)	38(31)	34(40)
	No	294(63)	158(62)	86(69)	50(60)
**Use NSAIDs**	Yes	164(45)	88(43)	47(47)	29(44)
	No	204(55)	115(57)	52(53)	37(56)
**Take Statins**	Yes	200(43)	114(45)	57(46)	29(35)
	No	263(57)	142(55)	67(54)	54(65)
**Home O**_**2**_	24 hours	41(9)	21(8)	12(10)	8(10)
	Only nighttime	10(2)	7(3)		3(4)
	No	414(89)	228(89)	113(90)	73(87)
**Use gas stove**	Yes	201(45)	107(43)	54(46)	40(48)
**Clinical Presentation**					
	**Pneumonia**	185 (41)	34 (14)	101 (86)	50 (62)
	**Non pneumonia (bronchitis)**	262 (59)	214 (86)	17 (14)	31 (38)
**Days between symptom onset and admission (Average)**		4.7	3.8	5.7	6

Distributions of pollutant concentrations for cases and controls, by lag time, are presented in S2 Table in S1 File. The highest correlations were observed between weekly concentrations of BC and AMP (r = 0.73), and NO_2_ and DC (r = 0.63)) (S3 Table in S1 File). Weaker correlations were observed between all other pairs of pollutants, temperature, and relative humidity, with the majority of correlations <0.5 except temperature and SO_2_ (r = −0.73).

**Fig 2:**
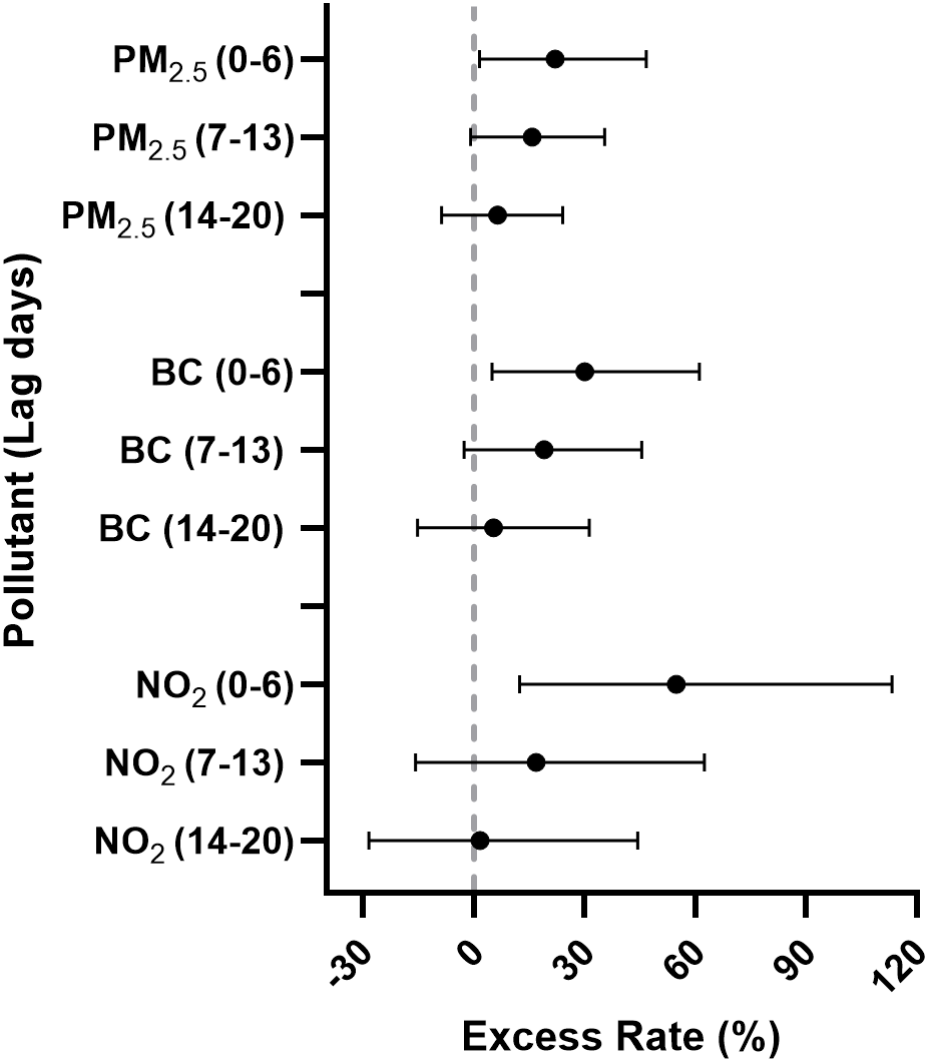
Excess rate of respiratory viral infection hospitalizations associated with interquartile range increases in PM_2.5_, BC, and NO_2_ concentrations, by lag time.

**Fig 3:**
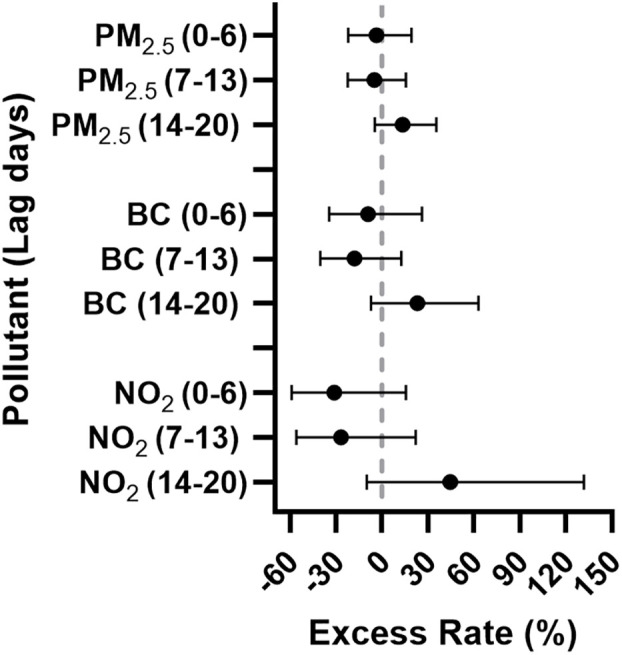
Excess rate of respiratory bacterial infection hospitalizations associated with interquartile range increases in PM_2.5_, BC, and NO_2_ concentrations, by lag time.

The excess rate of RVI hospitalization was generally largest for the traffic related pollutants in the 0–6 days lag period, next largest in the 7–13 days lag period, and null in the 14–20 lag period (S4 Table in S1 File). For example, each 2.6 µg/m^3^ increase in PM_2.5_ concentration in the 0–6 days prior to hospitalization was associated with a 22.1% increase in the rate of viral respiratory infection (95% CI: 1.6%, 46.7%). Similarly, a 30.0% increase (95% CI 5.0%, 61.1%) in the rate of RVI was associated with each 0.1 µg/m^3^ increase in BC concentration on lag days 0–6. NO_2_ and CO were also associated with an increased rate of RVI hospitalizations in the 14 days prior to admission (lag days 0–6 and 7–13; Fig 2), although increased CO concentrations were also associated with an increased rate of RVI in the 14–20 day lag period. The associations between RVI hospitalizations and other pollutants (DC, UFP, AMP, and SO_2_), but not O_3,_ generally had similar directions and magnitudes with PM_2.5_ and BC at all lag times (Fig 3).

Although not statistically significant, increased rates of RBI hospitalizations were associated with increases in PM_2.5_, BC, and NO_2_ concentrations in the 14–20 days prior to hospitalization (Fig 3, S4 Table in S1 File). Specifically, each 0.1 µg/m^3^ increase in BC concentration in the 14–20 days prior to hospitalization was associated with a 23.0% increase in the rate of RBI (95% CI: −7.3%, 63.1%), but decreased rates of RBI were associated with increased BC concentrations on lag days 0–6 and 7–13. Aside from an increased rate of RBI associated with increased concentrations of DC at the 14–20 lag days (marker of biomass), increased concentrations of other pollutants (UFP, AMP, SO2, and O_3_) were not associated with increased rates of RBI (S4 Table in S1 File).

Although not statistically significant, the excess rate of hospitalization for RVBI was largest at the 7–13 day lag period (S4 Table in S1 File). For each 0.1 µg/m^3^ increase in BC concentration in the 7–13 days prior to hospitalization, there was a 20.1% increase in the rate of RVBI (95% CI: --15.4%, 70.4%). The rate ratios were null or <1.0 at the other lag periods. Aside from PM_2.5_, NO_2_ and DC (rate ratios >1.0 on lag days 7–13, and PM_2.5_ and DC on lag days 0–6) and UFP (rate ratio >1.0 on lag days 14–20) the rate ratios for AMP, CO, SO_2_ and O_3_ were <1.0.

**Fig 4:**
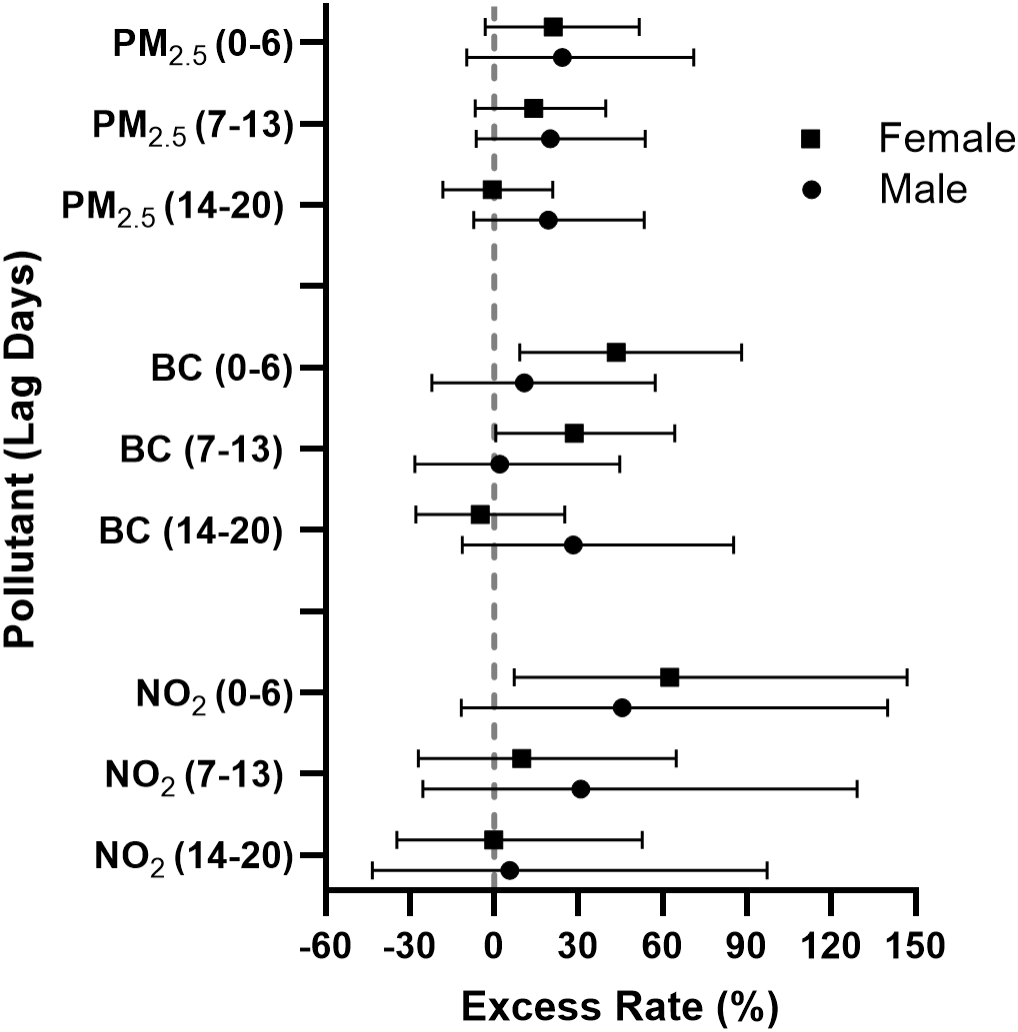
Excess rate of respiratory viral infection hospitalizations associated with interquartile range increases in PM_2.5_, BC, and NO_2_ concentrations by lag time, stratified by sex.

When stratifying by sex, females had a higher rate of hospitalization for RVI associated with increased BC concentrations compared to males at the 0–6 and 7–13 lag times, but a lower rate of hospitalizations than males at the 14–20 lag days. PM_2.5_ was also found to have higher rates of hospitalization for RVI in males at the 14–20 lag days than females (Fig 4). Each 0.1 µg/m^3^ increase in BC concentration in the previous 7 days (lag day 0–6) was associated with 43.4% (95% CI: 9.2%, 88.2%) and 10.8% (95% CI: −22.1%, 57.4%) increases in the rates of RVI in females and males, respectively. Although not statistically significant, both males and females had increased rates of hospitalization for RVI associated with increased NO_2_ on lag days 0–6 and a higher rate for males on lag days 7–13. No other consistent patterns of sex-specific differences across lag times were present (S5 Table in S1 File).

There were no consistent differences in rate ratios when stratifying by the Area deprivation index (ADI) (S6 Table in S1 File). However, when stratifying by distance from the monitor, the rate of hospitalization for RVI associated with IQR increases in BC and NO_2_ in the 1 week prior to hospitalization was greater for participants living over 5 miles from the monitor than those living 5 or less miles from the monitor (S6 Fig and S7 Table in S1 File). Although the lag day 0–6 results using the standard time-stratified case-crossover design had a smaller excess rate associated with each 3.7 ppb increase in NO_2_ concentration (21.5%; 95% CI = −11.3%, 66.4%) than the main analysis using the modified seasonal time-stratified design (54.8%; 95% CI: 12.4%, 113.3%), they were not significantly different (Table 2). PM_2.5_, BC, CO, SO_2_, AMP and UFP had a similar pattern of larger excess rates in the modified seasonal time-stratified design, but DC and O_3_ did not. We then examined whether the rate of RVI associated with increased PM_2.5_ and traffic related pollution concentrations on lag days 0–14 was different for the three smoking groups. The highest rate of hospitalization was present in patients who formerly smoked or those who never smoked compared to those who actively smoked (S7 Fig and S8 Table in S1 File). Finally, the rate of hospitalization was higher in patients with obstructive lung disease (COPD or asthma) than in those without at all periods for BC exposure and at the 0–6 and 7–13 lag periods for NO_2_ and DC exposure (S8 Fig and S9 Table in S1 File).

**Table 2:** Comparison of excess rates between two measurement methods among participants with viral infections.

Air pollutant	Lag days	Time-stratified design	N	IQR	Excess rate % (95% CI)	p-value
PM_2.5_ (µg/m^3^)	0-6	Novel Seasonal	256	2.6	22.1 (1.6, 46.7)	0.03
		Standard	256	2.6	16.3 (−5.0, 42.4)	0.14
BC (µg/m^3^)	0-6	Novel Seasonal	255	0.1	30.0 (5.0, 61.1)	0.02
		Standard	255	0.1	21.3 (−2.6, 51.1)	0.08
NO_2_ (ppb)	0-6	Novel Seasonal	254	3.7	54.8 (12.4, 113.3)	0.01
		Standard	254	3.7	21.5 (−11.3, 66.4)	0.23
CO (ppb)	0-6	Novel Seasonal	248	0.1	7.9 (−5.0, 22.5)	0.24
		Standard	248	0.1	−4.0 (−16.8, 10.6)	0.57
DC (µg/m^3^)	0-6	Novel Seasonal	255	0.1	9.9 (−8.8, 32.6)	0.32
		Standard	255	0.1	14.0 (−5.3, 37.3)	0.17
UFP (particles/cm^3^)	0-6	Novel Seasonal	229	1182.5	−1.4 (−20.9, 22.9)	0.90
		Standard	229	1182.5	−18.7 (−36.0, 3.1)	0.09
AMP (particles/cm^3^)	0-6	Novel Seasonal	229	278.6	14.8 (−8.8, 44.6)	0.24
		Standard	229	278.6	3.1 (−18.4, 30.5)	0.80
SO_2_ (ppb)	0-6	Novel Seasonal	256	0.1	8.1 (−14.2, 36.3)	0.51
		Standard	256	0.1	−6.2 (−28.6, 23.1)	0.64
O_3_ (ppm)	0-6	Novel Seasonal	250	0.01	−11.9 (−44.2, 39.3)	0.59
		Standard	250	0.01	−7.0 (−44.9, 57.1)	0.79

## Discussion

In this study of 465 participants who were admitted for respiratory infection, we observed an increased rate of RVI hospitalization associated with increased concentrations of traffic related pollutants (PM_2.5_, NO_2_, and BC) on lag days 0–6 and 7–13. In sensitivity analyses, females appeared to have a higher rate of hospitalization for RVI associated with increased BC compared to males on lag days 0–6 and 7–13. We also observed higher rates of RVI associated with air pollution in patients with COPD/asthma or those who never smoked or previously smoked compared to those who actively smoked. Though limited by imprecision, the largest rate of RBVI associated with increases in these traffic pollutant concentrations and DC concentrations were on lag days 7–13, while the largest rates of RBI associated with increased PM_2.5_, BC and NO_2_ concentrations were observed on lag days 14–20.

Our PM_2.5_/RVI results from lag days 0–6 (22.1%; 95% CI = 1.6%, 46.7%) are consistent with our previous work in New York State [27] from 2017–2019 (rescaled to an IQR = 2.6 µg/m^3^; Excess rate = 7.4%, 95% CI: 3.8%, 11.1%). In addition, a study examining the association between PM_2.5_ and positive testing for Influenza in a group of 4,229 adults over the age of 60 in Guangzhou, China observed only a 1.5% (95% CI: 0.5%, 2.8%) increase in the rate of a healthcare encounters for influenza per 2.6 µg/m^3^ in PM_2.5_ [28]. We also observed increased rates of hospitalization for RVI associated with black carbon, a measure of traffic pollution. A study of hospitals and clinics in Sichuan, China [8] reported an increased rate of healthcare encounters for influenza A (54.3%; 95% CI 32.9%, 79.1%) associated with each 3.7 ppb increase in NO_2_ concentrations (another marker of traffic pollution), which was consistent with the excess rate for hospitalization for RVI that we observed for the same 3.7 ppb increases in NO_2_ concentration on lag days 0–6 (54.8%; 95% CI: 12.4%−113.3%]). Both studies had small sample sizes (n = 836 for Li et al, n = 465 for our study) and the rate ratios for Influenza A or RVI were larger for NO_2_ than PM_2.5_. In a larger study of adults over 60 years of age (N = 4,229) in Guangzhou, China, each 3.7 ppb increase in NO_2_ concentration was associated with a 14.9% (95% CI: 8.9%, 22.0%) increase in the risk of a healthcare encounter for influenza [9]. Our results are consistent with this study as well, even though there were differences in the study populations (outpatient and inpatient in China vs. inpatient only in the current study), higher ambient pollution in China (mean [standard deviation] PM_2.5_ = 35.5 [16.0] µg/m^3^ in Guangzhou and 36.6 [22.2] µg/m^3^ in Sichuan, and different outcome ascertainment method [database review in China vs. direct enrollment in Rochester]).

Our study shows that with diligent outcome determination (clinical adjudication of every diagnosis of RVI, RBI and RVBI over 4 years) and its resultant lower degree of outcome misclassification and bias, it is possible to observe similar findings with 256 participants with RVI as we previously did with 6,981 participants [29] and understand how these differ from the lag patterns of RBI and RVBI. The time and expense of performing our current study is a helpful comparator to larger database driven studies which accept a greater degree of outcome misclassification in return for larger sample sizes. The similar inference drawn in our current study and prior studies of RVI supports the use of large data sets to assess large populations more economically. Though the direction of the associations between traffic related air pollution and RVI were similar comparing our novel seasonal time-stratified design to the standard case-crossover approach at the 0–6 lag time, the magnitude of the effect estimates for the novel approach were larger. While the novel seasonal approach shows potential for studying diseases that require lag times over 1 week, this seasonal modification to the case-crossover approach will require further validation.

In future studies, the use of broad infection definitions (such as ‘respiratory infection’ or ‘influenza-like-illness’) will be less able to advance our understanding when compared to studies using specific types of infection including viral, bacterial, coinfection and culture negative infections. Specifically, as the diagnosis of RSV infection in adults improves, there will be an opportunity to characterize the air pollution/RSV association in adults as well. Though the exploration of pollutant associations with specific viruses is important, caution must be used when considering combining novel respiratory diseases like SARS-CoV-2 (during the initial pandemic) with existing seasonal respiratory viruses in analyses. Of note, the substantial drop in non-COVID respiratory viral disease risk (23−94% decrease) during the early COVID-19 pandemic (2020−2021) also reduced the recruitment into this study for all types of infections [30]. Though many patients with SARS-CoV-2 infection had RVI alone or RVBI from bacterial superinfection, anyone with COVID-19 was excluded from this study as the pandemic dynamics (behavioral change, change in virulence) limited the ability to compare to the existing literature for RVI diagnoses [31]. As novel infections like SARS-CoV-2 become part of the normal respiratory viral infection season, including these in analyses of overall RVI should be more feasible.

Though the lag patterns in the RVI group were more robust than the imprecise RVBI and RBI patterns, the overall trends for RVBI and RBI can be hypothesis generating. For the traffic related pollutants (PM_2.5_, BC and NO_2_), the rate of hospitalization for RVI was largest in the earlier lag times (0–6 and 7–13 days), while the rate of hospitalization for RBI was generally largest at the later lag times (lag days 14–20). Combined infection was observed to have the largest effect estimates in the 7–13 lag day period. A reasonable hypothesis would be that TRAP may increase the risk of severe RVI (requiring hospitalization) by altering aspects of innate immunity in the 1–13 days prior to and during the initial RVI (S2 Fig in S1 File). The relationship between TRAP and RBI and RVBI is less clear. First, it is possible that for patients with RBI, the cell based (adaptive) response was impaired from exposure at a longer lag time (14–20 days) prior to admission [32]. Alternatively the lag period pattern for RBI may support the hypothesis that the majority of RBI may be preceded by a RVI (Supplemental Text and S3 Fig in S1 File), which may be due to RVIs impairing macrophage function and a direct enhancement of bacterial virulence by viruses binding to bacteria [33].

While females had a higher rate of hospitalization for RVI associated with increased concentrations of BC compared to males in the early lag periods, the rates of hospitalization for males were higher in the longest lag time (14–20) for BC and PM_2.5_. In a prior study of New York State patients with influenza [29], males had a similar rate ratio as females in 2017–2019 compared to 2014–2016 (when the rate of hospitalization for males was smaller than females). Of note, BC, NO_2_, and other pollutants that were not included in this prior study were included in our current analysis. While there is not a consistent sex-specific difference for PM_2.5_ (both sexes had a higher rate of hospitalization for RVI than in the prior NYS study) in our current analysis, females appear to have a greater response to BC than males in early lag periods (Fig 4). The underlying mechanism of sex-specific differences in RVI response to pollution is still an area of active research as these differences have been reported in some [11,27,34], but not all studies of pollution and respiratory infection [2,9]. The difference in lag pattern (rates of RVI hospitalization for females higher in 0–13 period and higher for males in the 14–20 period), will need further study to determine what aspect of the immune system or other sex related factors could explain this difference.

Combustible tobacco smoking can increase the risk of respiratory infection by impairing host immune defense including delayed mucociliary clearance and disruption of the airway epithelium [13]. In this study, we explored whether our findings of air pollution/RVI associations were robust to residual confounding from cigarette smoking. Though our stratified analyses were limited by imprecision, we generally observed larger air pollution/RVI rate ratios in patients who never smoked (or formerly smoked) when compared with patients who were actively smoking (S7 Fig and S8 Table in S1 File). The pattern of rate ratios for people who formerly smoked, and people who never smoked appeared similar in magnitude and direction to the rate ratios observed for traffic-related pollution (e.g., NO_2_, BC) and RVI. It is more likely that patients who were actively smoking had a decreased response to air pollution than those who formerly smoked (or never smoked) due to cigarette smoking being a more intense exposure than ambient pollution. Specifically, it is possible that we could not observe an additional risk of RVI hospitalization from air pollution in the smoking population as they already had an elevated risk of RVI hospitalization from smoking itself. The current literature would not support the alternative explanation that cellular adaptation from cigarette smoke exposure would be protective against air pollution exposure [35]. Residual confounding within this stratified analysis and the potential for behavioral differences between those who actively smoke and others (e.g., diet, exercise) may also contribute to the observed patterns. We also observed a higher rate of hospitalization for RVI in patients with obstructive lung disease (COPD or asthma) associated with traffic related pollution (BC and NO_2_) and wood smoke (DC). As the potential for smoking or pre-existing conditions (COPD and asthma) to modify the association between air pollution and RVI continues to be studied, we will also need to explore the potential for synergistic harm between combustion related pollution, active smoking, and pre-existing lung disease in patients with RVI.

To characterize other environmental and societal exposures, we explored effect modification by levels of ADI in Rochester. Considering environmental justice when studying the health effects of air pollution is a critical area of public health research [36,37]. Despite the lack of findings within the ADI stratification, we did observe a higher rate of hospitalization for RVI associated with TRAP for individuals living over 5 miles from the air quality monitor. The area between the 5 and 15 miles radius includes more census block groups with high ADI than the area under 5 miles (S5 Fig in S1 File). Use of central-site pollutant concentrations in epidemiology studies is limited by varying degrees of exposure misclassification and downward bias in effect estimates (i.e., rate ratios in this study) [38]. These sensitivity analyses, although expected to show larger rate ratios for those living closer (≤5 miles) to the monitoring station compared to those living farther away (>5 miles), may instead represent different patterns of daily personal activities relative to TRAP pollutant exposures. For example, the monitored TRAP concentrations at the monitor for study participants living near the monitoring station may represent only residential TRAP exposures, but not the TRAP exposure at work or other activity locations. However, for a portion of the patients living farther from the monitoring station (i.e., in a more densely population urban center), TRAP exposures from the monitoring site may better represent both residential and work/other location TRAP exposures. These potential explanations will need to be formally examined in future work. Spatiotemporal models using monitored values across the city will be developed and should provide rate ratio estimates with lower degrees of exposure misclassification and downward bias in future RVI epidemiology studies.

There are several limitations to consider in this study. This study used a novel modified time-stratified case-crossover design including three-month seasons in which to match case and control periods for each matched set. This approach allowed us to examine associations with lagged pollutant concentrations longer than lag days 0–6. While our sensitivity analysis observed that the rate ratios were larger for the new seasonal time-stratified case-crossover design when compared to the standard time stratified case-crossover design, the direction of the effect was the same between the two methods and rate ratios were not significantly different. Though this new seasonal time stratified case-crossover design overcomes limitations of the standard case-crossover design, we will need to continue to validate its use. Furthermore, given the need to compare this modification to the standard case-crossover design in prior studies, we retained a weeklong lag structure (days 0–6, 7–13, 14–20) rather than employing a distributed lag model.

The generalizability of this study is limited to hospitalized patients with respiratory infection. It would not be directly applicable to patients with respiratory infection who are treated in the outpatient clinics or ambulatory settings. The focus on ambient (outdoor) air pollution in this study limits the application of our findings to studies of indoor air pollution, though the stratification by smoking may be hypothesis generating (as smoking is one of the most important personal pollution exposures). Given the rigorous effort to minimize outcome misclassification by enrolling only patients with the highest certainty of respiratory infection diagnoses (after adjudication by 4 physicians), the size of the population of this study was relatively low (n = 465). In particular, this likely contributed to the lack of precision in the combined infection (RVBI) group (n = 84). Given the low sample size for the stratification analyses, these should be considered exploratory and will require replication. While the pragmatic seasonal modification including lag weeks (0–6, 7–13 and 14–20 days) allows for comparison to prior studies and provides dimension reduction in terms of models, we were not able to report fine resolution of individual lag days as in a distributed lag model. Though we focused our analysis on individual pollutant models to characterize the novel seasonal modification to the standard case-crossover design, the individual pollutant assessments can inform future multipollutant models with this same seasonal approach. Though the restriction of participants to those living less than 15 miles from the central air pollution monitoring site reduced the number of our study participants, this restriction also reduced exposure misclassification. Finally, our study was conducted in a relatively low pollution area, limiting direct comparison with higher pollution areas around the world. However, given that the average PM_2.5_ concentration in Rochester, NY is below the PM_2.5_ national ambient air quality standards (NAAQS), research conducted in this setting is policy relevant in the association between air pollution and health effects are still observed at concentrations below the NAAQS standard.

## Conclusions

In a study examining the association between ambient air pollution and the rate of hospitalization for respiratory infections, we observed stronger associations between increased PM_2.5_, NO_2_ and BC concentrations and the rate of RVI, than with RBI and RVBI during the two weeks prior to admission (lag days 0–6, 7–13). During these two weeks, we observed stronger associations in females than males for BC exposure and stronger associations for patients with COPD/asthma and TRAP exposure. Future work is needed to determine the underlying mechanisms driving the difference in RVI/TRAP effects and the subgroups who appear to be uniquely affected.

## Supporting information

S1 File Figures S1–S8 and Tables S1–S9 can be found in the supporting information file. (DOCX)
