# Supplementary material for: Triggering of viral and bacterial respiratory infection hospitalizations by traffic pollution exposure in a cohort of hospitalized adults
Source: PLoS One. 2026 Jul 13;21(7):e0352323. doi: 10.1371/journal.pone.0352323 (PMC13362091; doi:10.1371/journal.pone.0352323)
Supplement: S1 File — (DOCX) [file pone.0352323.s001.docx]

**Supplement: Triggering of viral and bacterial respiratory infection hospitalizations by traffic pollution exposure in a cohort of hospitalized adults**

Authors:

Daniel P. Croft,^1,2^ Md Rayhanul Islam,^2^ Kelly Thevenet-Morrison,^2^ Carl J. Johnston,^1^ Mark J. Utell,^1,2^ Philip K. Hopke,^2,3^ Steve N. Georas,^1,2^ Augusto A. Litonjua,^4^ Derick R. Peterson,^5^ Soumyaroop Bhattacharya,^4^ Andrea Baran,^5^ Chinyi Chu,^4^ Anthony Corbett, ^6^ Michael Peasley,^7^ Angela R. Branche,^7^ Edward E. Walsh,^7^ Matthew N. McCall,^5,7^ Sally W. Thurston,^2,5^ Ann R. Falsey,^7^ Thomas J. Mariani,^4,8^ David Q. Rich^1,2^

1. Department of Medicine, Pulmonary and Critical Care Medicine Division. University of Rochester Medical Center, Rochester, New York, United States of America
2. Department of Environmental Medicine and Public Health Sciences, Rochester, New York, United States of America
3. Institute for a Sustainable Environment, and Center for Air Resources Engineering and Science, Clarkson University, Potsdam, New York, United States of America
4. Department of Pediatrics, University of Rochester Medical Center, Rochester, New York, United States of America
5. Department of Biostatistics and Computational Biology, University of Rochester Medical Center, Rochester, New York, United States of America
6. Clinical and Translational Science Institute, University of Rochester Medical Center, Rochester, New York, United States of America
7. Department of Medicine, Infectious Diseases Division. University of Rochester Medical Center, Rochester, New York, United States of America
8. Department of Biomedical Genetics. University of Rochester Medical Center, Rochester, New York, United States of America

All authors are located in the United States of America.

**Supplemental Text on timing of pollution exposure and respiratory infection:**

The most relevant lag period for the association between air pollution and respiratory viral infection is not known. In our study we observed the strongest effect estimates at the zero to 6 lag day, which would roughly correspond to a period during active infection (possibly spanning the end of the incubation period until hospitalization with worsened symptoms (Figure S2).

In a scenario where a viral RVI precedes RBI, air pollution exposure could contribute to an increased susceptibility to a RVI (at 16 days prior to admission for example) (Figure S3). Next, the patient would become ill with the RVI (lag days 16 to 9) and finally suffer from a bacterial superinfection of their prior RVI (lag day 9) until they are symptomatic enough to present at day 0 (our study found that patients presented for RBI around 6 days after symptoms started). The fact that the RBVI (combined infection) group had the highest rate of hospitalization associated with increased air pollutant concentration in lag days 7-13 could support the hypothesis that these individuals with RBVI proceeding along a similar clinical course (tempo of illness) as the patients with RBI, except that the combined infection group presented to the hospital earlier than the RBI group. Therefore, on testing, we may have detected the original viral infection (that had yet to be fully cleared) and a new superinfection with a bacterial organism. If this is the case, it is possible that the most meaningful increased risk related to pollution is the risk of the initial RVI, and it may be possible that air pollution is less strongly associated with RBI on its own.


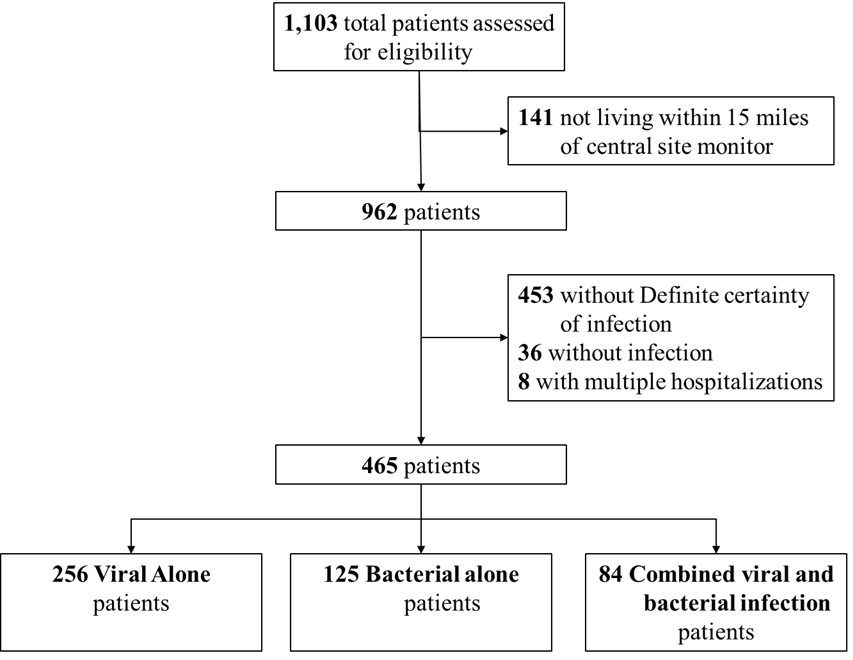


**S1 Fig.** CONSORT flow diagram.


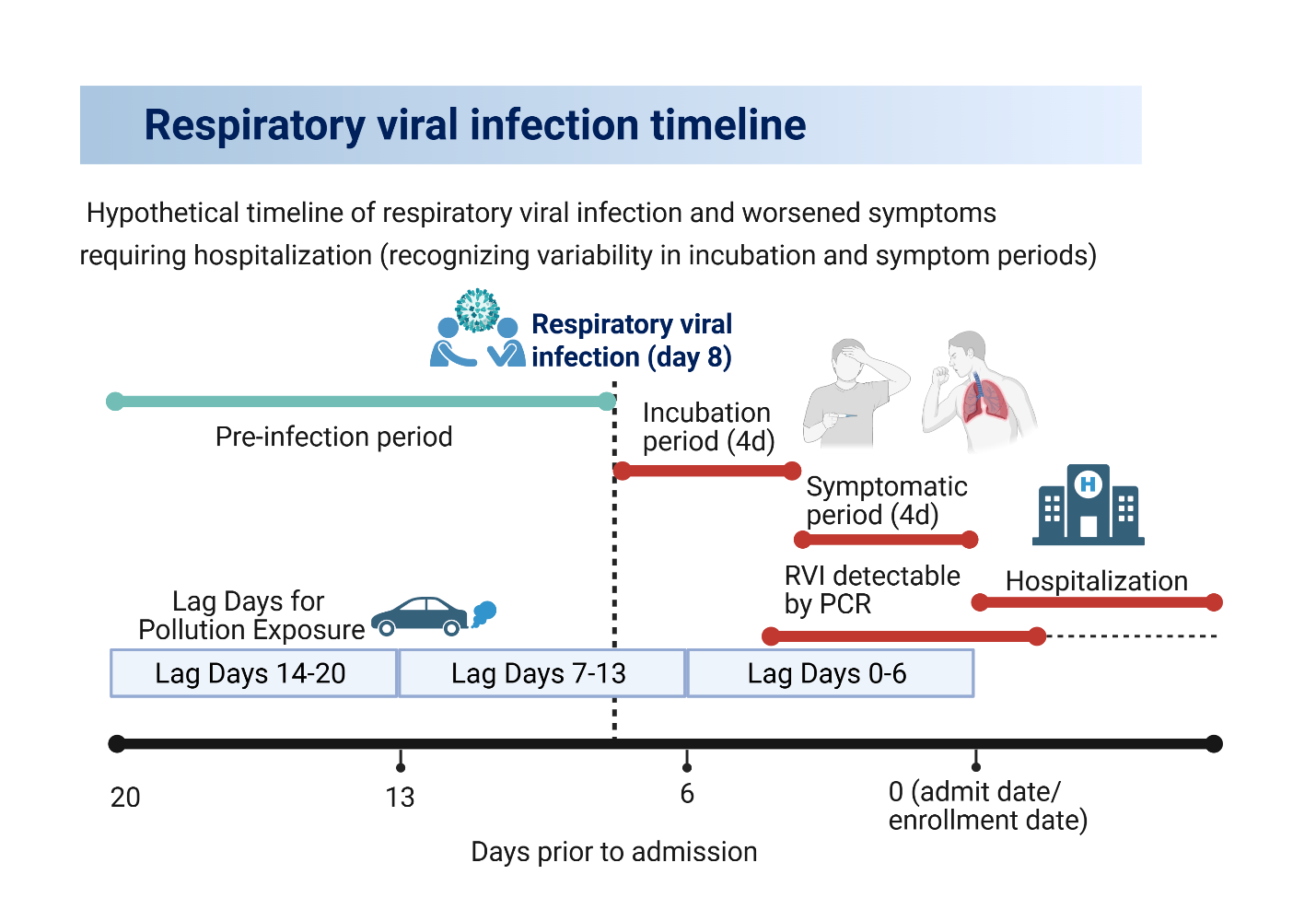


**S2 Fig.** Hypothetical Respiratory Viral Infection Timeline. Created with BioRender template.


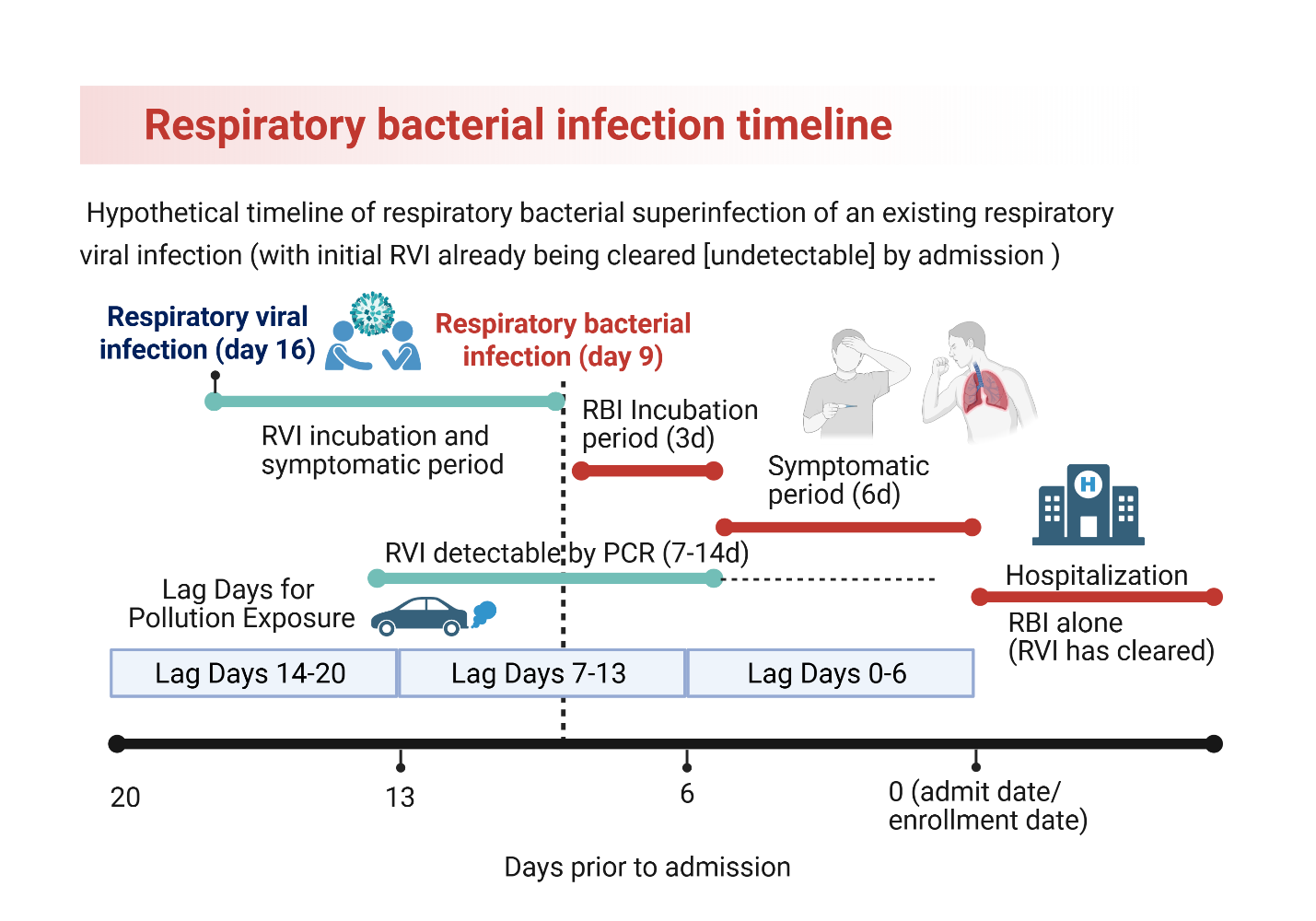


**S3 Fig**. Hypothetical respiratory bacterial infection timeline. Created with BioRender template.


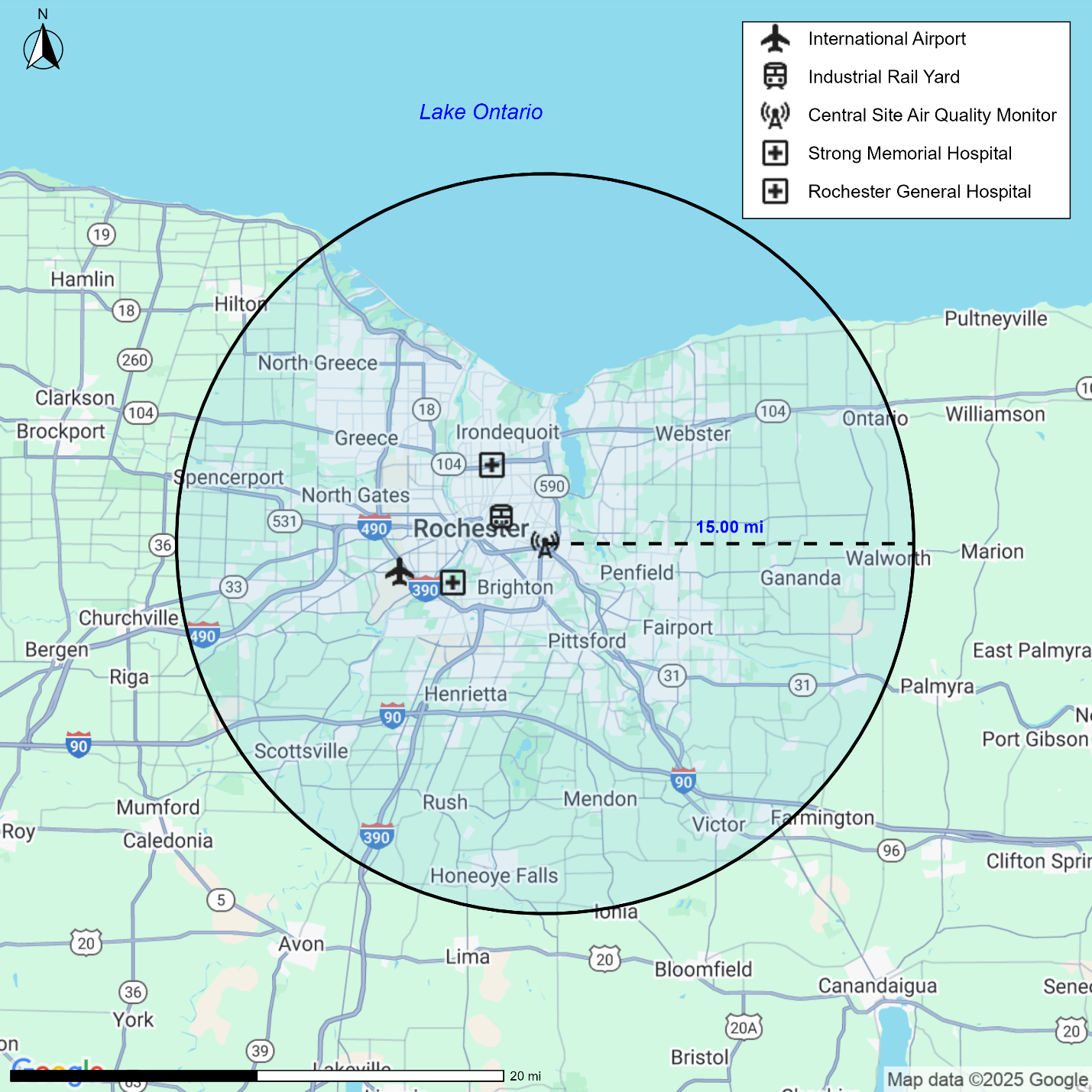


**S4 Fig .** Map of the Rochester, NY area and the 5- and 15-mile radius around the central site monitor from which our population was enrolled.


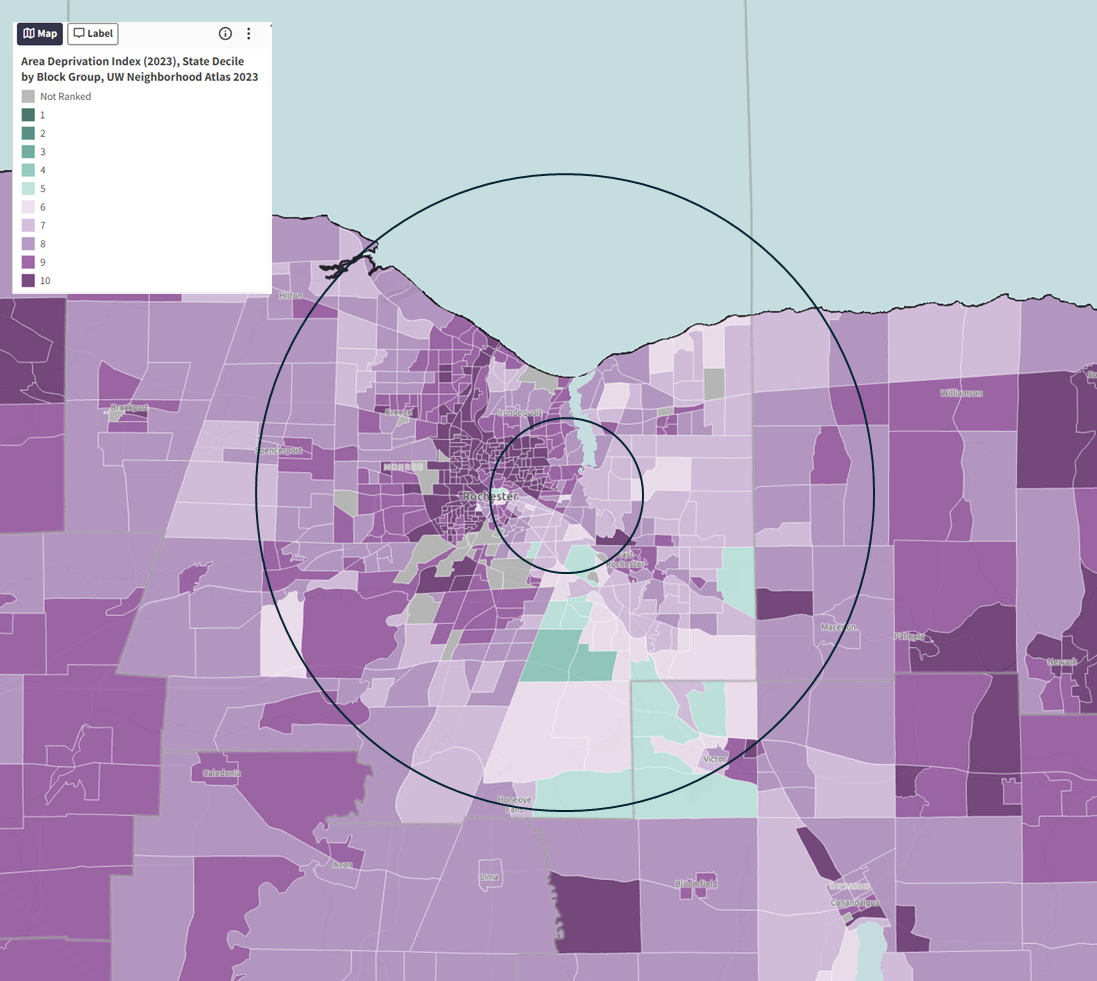


**S5 Fig**. Area deprivation map of Rochester with 5- and 15- mile radius around the central site air quality monitor (Darkest purple has highest deprivation). Source: Rochhealthdata.org.

**S6 Fig.** Excess rate of viral infections related hospital admissions associated with interquartile range increases (IQR) in air pollutants concentration, stratified by distance from the central air quality monitor by lag time. The double forward slash on NO_2_ at 0-6 lag period indicates the confidence interval extends past the upper limit of the x axis.

**
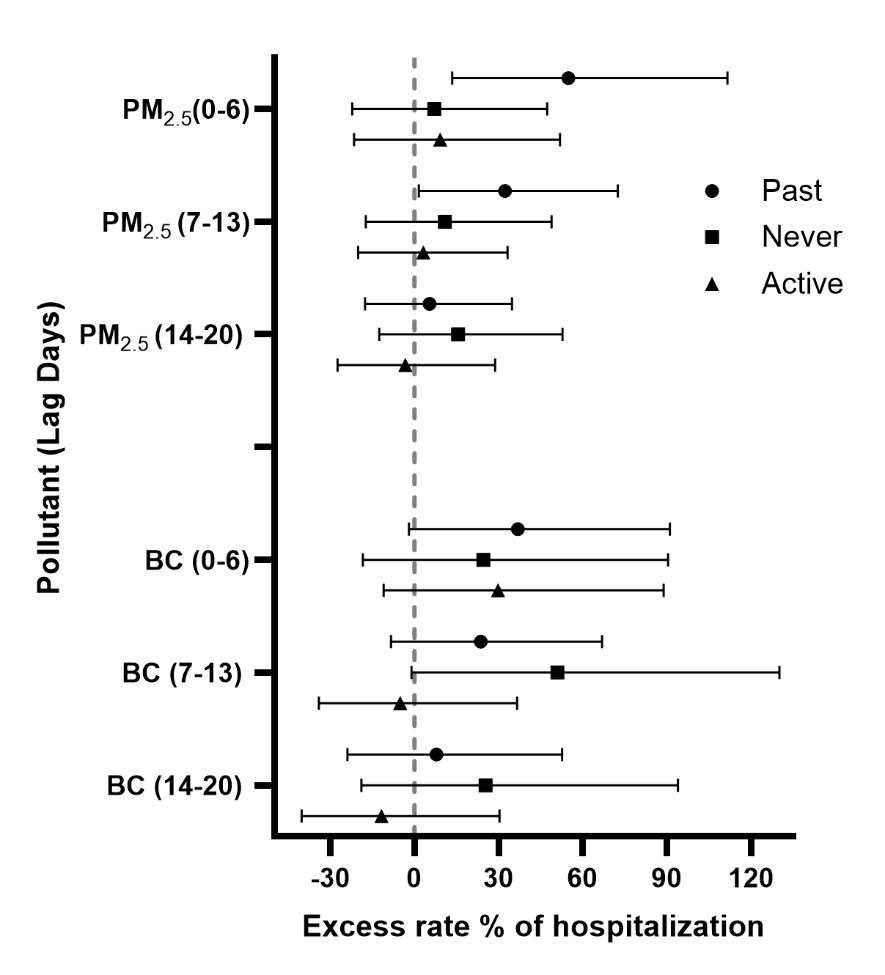
**

**S7 Fig**. Association between pollutants and respiratory viral infection hospitalizations stratified by smoking.


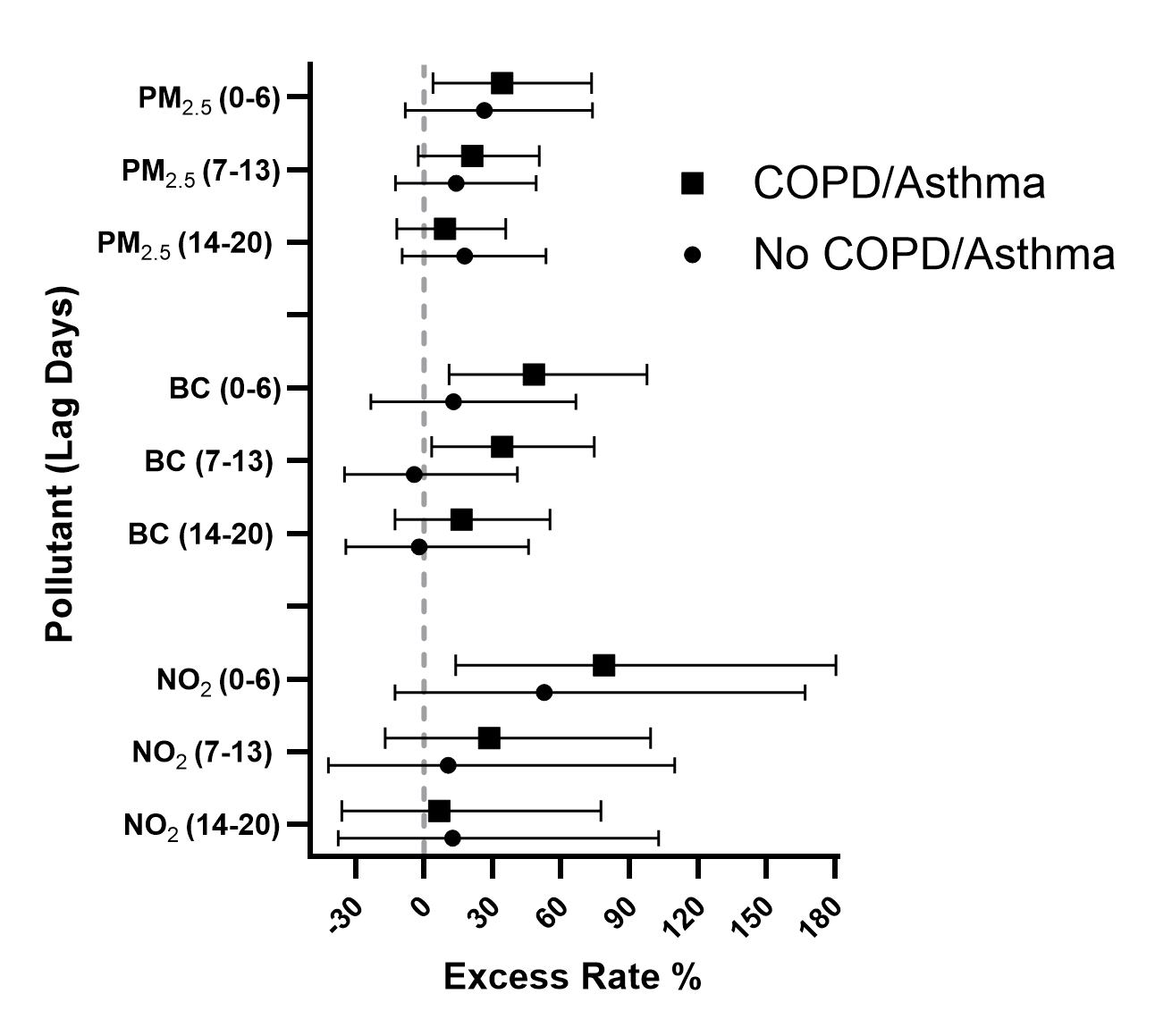


**S8 Fig.**. Lung disease specific excess rate of viral infections related hospital admissions associated with interquartile range increases (IQR) in air pollutants concentration, by lag time

**S1 Table.** Viral and Bacterial pathogens

|  | **Pathogens** | **Positive (%)** |
| --- | --- | --- |
| **Viral (n=256)** | Adenovirus | 5(2.0) |
|  | Coronavirus (not SARS-CoV-2) | 6(2.3) |
|  | Influenza A | 90(35.2) |
|  | Influenza B | 8(3.1) |
|  | Human metapneumovirus | 23(9.0) |
|  | Parainfluenza virus | 23(9.0) |
|  | Rhinovirus | 75(29.3) |
|  | Respiratory Syncytial Virus | 31(12.1) |
| **Bacterial (n=125)** | E. coli | 4 (3.2) |
|  | Haemophilus influenzae | 14 (11.2) |
|  | Klebsiella pneumoniae | 4 (3.2) |
|  | Legionella | 10 (8.0) |
|  | Moraxella catarrhalis | 6 (4.8) |
|  | Mycoplasma | 7 (5.6) |
|  | Pseudomonas aeruginosa | 4 (3.2) |
|  | Staphylococcus aureus | 7 (5.6) |
|  | Streptococcus | 13 (10.4) |

**S2 Table.** Weekly ambient air pollution concentrations for all participants, stratified by case and control days.

| **Pollutants** | **Lag period prior to diagnosis** | **Case Days**  **Median (IQR)** | **Control Days**  **Median (IQR)** |
| --- | --- | --- | --- |
| PM_2.5_  (µg/m^3^) | 0-6 | 6.52(2.51) | 6.32(2.6) |
|  | 7-13 | 6.27(2.36) | 6.23(2.28) |
|  | 14-20 | 6.48(2.46) | 6.23(2.18) |
| BC  (µg/m^3^) | 0-6 | 0.31(0.14) | 0.31(0.13) |
|  | 7-13 | 0.3(0.13) | 0.31(0.13) |
|  | 14-20 | 0.31(0.13) | 0.31(0.13) |
| NO_2_  (ppb) | 0-6 | 6.71(3.4) | 6.14(3.73) |
|  | 7-13 | 6.56(3.74) | 6.46(3.85) |
|  | 14-20 | 6.86(3.54) | 6.48(3.73) |
| CO  (ppb) | 0-6 | 0.23(0.05) | 0.22(0.06) |
|  | 7-13 | 0.22(0.06) | 0.21(0.06) |
|  | 14-20 | 0.22(0.07) | 0.22(0.06) |
| DC  (µg/m^3^) | 0-6 | 0.11(0.09) | 0.1(0.09) |
|  | 7-13 | 0.11(0.08) | 0.11(0.09) |
|  | 14-20 | 0.11(0.09) | 0.1(0.09) |
| UFP (particles/cm^3^) | 0-6 | 3999.27(1184.51) | 4071.31(1195.23) |
|  | 7-13 | 4057.78(1178.3) | 4043.79(1139.98) |
|  | 14-20 | 3991.32(1147.05) | 4071.31(1189.03) |
| AMP (particles/cm^3^) | 0-6 | 585.92(288.34) | 585.92(268.73) |
|  | 7-13 | 562.05(272.08) | 565.4(282.7) |
|  | 14-20 | 557.78(282.2) | 579.82(261.5) |
| SO_2_  (ppb) | 0-6 | 0.13(0.12) | 0.12(0.14) |
|  | 7-13 | 0.12(0.12) | 0.12(0.13) |
|  | 14-20 | 0.13(0.14) | 0.12(0.13) |
| O_3_  (ppm) | 0-6 | 0.03(0.01) | 0.03(0.01) |
|  | 7-13 | 0.03(0.01) | 0.03(0.01) |
|  | 14-20 | 0.03(0.01) | 0.03(0.01) |
| Temperature (°F) | 0-6 | 45.51(23.36) | 47.91(28.71) |
|  | 7-13 | 44.36(27.33) | 46.94(30.55) |
|  | 14-20 | 43.46(24.95) | 47.56(30.31) |
| Relative humidity (%) | 0-6 | 66.9(11.47) | 68.32(11.59) |
|  | 7-13 | 68.51(10.22) | 68.51(11.64) |
|  | 14-20 | 68.43(10.52) | 68.24(10.78) |

**S3 Table.** Correlation coefficient among weekly (lag days 0-6) pollutant concentrations, temperatures and relative humidities.

|  | PM_2.5_ | BC | NO_2_ | CO | DC | UFP | AMP | SO_2_ | O_3_ | Temp |
| --- | --- | --- | --- | --- | --- | --- | --- | --- | --- | --- |
| BC | 0.45 |  |  |  |  |  |  |  |  |  |
| NO_2_ | 0.47 | 0.18 |  |  |  |  |  |  |  |  |
| CO | 0.22 | 0.34 | 0.13 |  |  |  |  |  |  |  |
| DC | 0.50 | 0.29 | 0.63 | 0.13 |  |  |  |  |  |  |
| UFP | -0.14 | 0.49 | 0.03 | 0.25 | 0.03 |  |  |  |  |  |
| AMP | 0.40 | 0.73 | 0.33 | 0.50 | 0.33 | 0.50 |  |  |  |  |
| SO_2_ | 0.40 | 0.11 | 0.45 | -0.02 | 0.29 | -0.18 | 0.47 |  |  |  |
| O_3_ | -0.20 | -0.23 | -0.29 | -0.01 | 0.05 | -0.01 | 0.05 | -0.18 |  |  |
| Temperature | -0.17 | 0.40 | -0.43 | 0.39 | 0.39 | 0.19 | -0.05 | -0.73 | -0.41 |  |
| Relative humidity | 0.18 | -0.03 | 0.04 | -0.24 | -0.11 | -0.66 | 0.07 | 0.10 | -0.01 | -0.15 |

**S4 Table.** Excess rate of respiratory viral, bacterial, and combined viral/bacterial hospitalizations associated with interquartile range increases (IQR) in PM_2.5_ concentration, by infection type and lag time.

| **Outcome** | **Air pollutants** | **Lag** | **N** | **IQR** | **Excess Rate %**  **(95% CI)** | **p-value** |
| --- | --- | --- | --- | --- | --- | --- |
| Viral | PM_2.5_  (µg/m^3^) | 0-6 | 256 | 2.6 | 22.1 (1.6, 46.7) | 0.03 |
|  |  | 7-13 | 256 | 2.2 | 15.8 (-0.9, 35.4) | 0.07 |
|  |  | 14-20 | 250 | 2.2 | 6.4 (-8.7, 24.1) | 0.43 |
|  | BC  (µg/m^3^) | 0-6 | 255 | 0.1 | 30.0 (5.0, 61.1) | 0.02 |
|  |  | 7-13 | 256 | 0.1 | 19.1 (-2.6, 45.5) | 0.09 |
|  |  | 14-20 | 256 | 0.1 | 5.4 (-15.3, 31.3) | 0.64 |
|  | NO_2_  (ppb) | 0-6 | 254 | 3.7 | 54.8 (12.4, 113.3) | 0.01 |
|  |  | 7-13 | 253 | 3.7 | 16.9 (-15.8, 62.4) | 0.35 |
|  |  | 14-20 | 255 | 3.8 | 1.7 (-28.4, 44.4) | 0.93 |
|  | CO  (ppb) | 0-6 | 248 | 0.1 | 7.9 (-5.0, 22.5) | 0.24 |
|  |  | 7-13 | 252 | 0.1 | 15.9 (2.3, 31.2) | 0.02 |
|  |  | 14-20 | 248 | 0.1 | 18.1 (-0.6, 40.3) | 0.06 |
|  | DC  (µg/m^3^) | 0-6 | 255 | 0.1 | 9.9 (-8.8, 32.6) | 0.32 |
|  |  | 7-13 | 256 | 0.1 | 1.8 (-15.5, 22.7) | 0.85 |
|  |  | 14-20 | 256 | 0.1 | -19.6 (-36.1, 1.2) | 0.06 |
|  | UFP (particles/cm^3^) | 0-6 | 229 | 1182.5 | -1.4 (-20.9, 22.9) | 0.90 |
|  |  | 7-13 | 211 | 1170.8 | 8.5 (-12.8, 35.1) | 0.46 |
|  |  | 14-20 | 220 | 1140.5 | 3.0 (-18.8, 30.6) | 0.81 |
|  | AMP (particles/cm^3^) | 0-6 | 229 | 278.6 | 14.8 (-8.8, 44.6) | 0.24 |
|  |  | 7-13 | 211 | 269.8 | 11.4 (-11.6, 40.4) | 0.36 |
|  |  | 14-20 | 220 | 280.5 | -5.8 (-25.7, 19.4) | 0.62 |
|  | SO_2_  (ppb) | 0-6 | 256 | 0.1 | 8.1 (-14.2, 36.3) | 0.51 |
|  |  | 7-13 | 256 | 0.1 | 7.3 (-12.4, 31.4) | 0.50 |
|  |  | 14-20 | 256 | 0.1 | 11.1 (-12.0, 40.3) | 0.37 |
|  | O_3_  (ppm) | 0-6 | 250 | 0.01 | -11.9 (-44.2, 39.3) | 0.59 |
|  |  | 7-13 | 250 | 0.01 | 3.0 (-31.8, 55.4) | 0.89 |
|  |  | 14-20 | 248 | 0.01 | -12.2 (-43.6, 36.7) | 0.56 |
| Bacterial | PM_2.5_  (µg/m^3^) | 0-6 | 125 | 2.6 | -3.6 (-22.0, 19.2) | 0.73 |
|  |  | 7-13 | 125 | 2.2 | -5.1 (-22.2, 15.8) | 0.60 |
|  |  | 14-20 | 123 | 2.2 | 13.6 (-4.8, 35.4) | 0.16 |
|  | BC  (µg/m^3^) | 0-6 | 122 | 0.1 | -9.0 (-34.3, 26.1) | 0.57 |
|  |  | 7-13 | 122 | 0.1 | -17.8 (-40.1, 12.7) | 0.22 |
|  |  | 14-20 | 122 | 0.1 | 23.0 (-7.3, 63.1) | 0.15 |
|  | NO_2_  (ppb) | 0-6 | 124 | 3.7 | -31.2 (-59.0, 15.7) | 0.16 |
|  |  | 7-13 | 124 | 3.7 | -26.6 (-55.8, 22.0) | 0.23 |
|  |  | 14-20 | 123 | 3.8 | 44.5 (-9.8, 131.6) | 0.13 |
|  | CO  (ppb) | 0-6 | 123 | 0.1 | 6.6 (-5.9, 20.7) | 0.32 |
|  |  | 7-13 | 122 | 0.1 | -10.3 (-25.5, 8.1) | 0.25 |
|  |  | 14-20 | 117 | 0.1 | -3.4 (-14.6, 9.3) | 0.58 |
|  | DC  (µg/m^3^) | 0-6 | 122 | 0.1 | -23.4 (-45.2, 7.3) | 0.12 |
|  |  | 7-13 | 122 | 0.1 | 2.6 (-25.1, 40.6) | 0.87 |
|  |  | 14-20 | 122 | 0.1 | 14.9 (-16.7, 58.4) | 0.40 |
|  | UFP (particles/cm^3^) | 0-6 | 105 | 1182.5 | 5.9 (-22.8, 45.2) | 0.72 |
|  |  | 7-13 | 103 | 1170.8 | -27.3 (-45.3, -3.4) | 0.03 |
|  |  | 14-20 | 110 | 1140.5 | -3.7 (-27.7, 28.2) | 0.80 |
|  | AMP (particles/cm^3^) | 0-6 | 105 | 278.6 | -13.2 (-38.5, 22.4) | 0.42 |
|  |  | 7-13 | 103 | 269.8 | -27.4 (-47.7, 0.7) | 0.05 |
|  |  | 14-20 | 110 | 280.5 | -19.4 (-42.6, 13.2) | 0.21 |
|  | SO_2_  (ppb) | 0-6 | 124 | 0.1 | -10.0 (-32.4, 20.0) | 0.47 |
|  |  | 7-13 | 124 | 0.1 | -26.7 (-45.9, -0.7) | 0.04 |
|  |  | 14-20 | 124 | 0.1 | -14.7 (-35.4, 12.5) | 0.26 |
|  | O_3_  (ppm) | 0-6 | 123 | 0.01 | -23.5 (-57.9, 39.3) | 0.38 |
|  |  | 7-13 | 125 | 0.01 | -40.3 (-67.0, 8.0) | 0.09 |
|  |  | 14-20 | 124 | 0.01 | -44.2 (-69.6, 2.7) | 0.06 |
| Bacterial and Viral | PM_2.5_  (µg/m^3^) | 0-6 | 84 | 2.6 | 17.8 (-14.2, 61.8) | 0.31 |
|  |  | 7-13 | 84 | 2.2 | 29.2 (-1.0, 68.5) | 0.06 |
|  |  | 14-20 | 82 | 2.2 | 0.1 (-26.1, 35.4) | 1.00 |
|  | BC  (µg/m^3^) | 0-6 | 84 | 0.1 | 10.6 (-23.6, 60.2) | 0.59 |
|  |  | 7-13 | 84 | 0.1 | 20.1 (-15.4, 70.4) | 0.30 |
|  |  | 14-20 | 84 | 0.1 | 2.1 (-30.4, 49.8) | 0.92 |
|  | NO_2_  (ppb) | 0-6 | 84 | 3.7 | -10.3 (-47.9, 54.5) | 0.69 |
|  |  | 7-13 | 84 | 3.7 | 31.9 (-22.4, 124.2) | 0.31 |
|  |  | 14-20 | 84 | 3.8 | 2.1 (-41.9, 79.2) | 0.94 |
|  | CO  (ppb) | 0-6 | 83 | 0.1 | -18.6 (-45.3, 21.2) | 0.31 |
|  |  | 7-13 | 83 | 0.1 | -5.5 (-33.7, 34.7) | 0.75 |
|  |  | 14-20 | 82 | 0.1 | -8.7 (-33.0, 24.4) | 0.56 |
|  | DC  (µg/m^3^) | 0-6 | 84 | 0.1 | 22.7 (-12.7, 72.7) | 0.24 |
|  |  | 7-13 | 84 | 0.1 | 33.4 (-4.9, 87.2) | 0.10 |
|  |  | 14-20 | 84 | 0.1 | -1.7 (-31.7, 41.6) | 0.93 |
|  | UFP (particles/cm^3^) | 0-6 | 75 | 1182.5 | -23.6 (-47.1, 10.5) | 0.15 |
|  |  | 7-13 | 71 | 1170.8 | -20.7 (-47.2, 19.2) | 0.26 |
|  |  | 14-20 | 73 | 1140.5 | 2.0 (-28.4, 45.4) | 0.91 |
|  | AMP (particles/cm^3^) | 0-6 | 75 | 278.6 | 2.2 (-33.2, 56.5) | 0.92 |
|  |  | 7-13 | 71 | 269.8 | -15.0 (-45.8, 33.4) | 0.48 |
|  |  | 14-20 | 73 | 280.5 | -19.6 (-48.1, 24.5) | 0.33 |
|  | SO_2_  (ppb) | 0-6 | 84 | 0.1 | -22.3 (-50.1, 21.2) | 0.27 |
|  |  | 7-13 | 84 | 0.1 | -4.0 (-33.3, 38.3) | 0.83 |
|  |  | 14-20 | 84 | 0.1 | -1.7 (-34.0, 46.4) | 0.93 |
|  | O_3_  (ppm) | 0-6 | 82 | 0.01 | -51.8 (-79.0, 11.0) | 0.09 |
|  |  | 7-13 | 83 | 0.01 | -65.3 (-84.3, -23.1) | 0.01 |
|  |  | 14-20 | 82 | 0.01 | -36.4 (-71.0, 39.3) | 0.26 |

**S5 Table: Sex specfic excess rate of viral respiratory infection hospital admissions associated with interquartile range increases (IQR) in PM2.5 concentration, by lag time.**

| **Air pollutants** | **Population Characteristics** | **Lag** | **N** | **IQR** | **Excess Rate %**  **(95% CI)** | **p-value** |
| --- | --- | --- | --- | --- | --- | --- |
| PM_2.5_ | Female | 0-6 | 162 | 2.6 | 21.1 (-3.2, 51.6) | 0.09 |
|  |  | 7-13 | 162 | 2.2 | 14.2 (-6.7, 39.7) | 0.20 |
|  |  | 14-20 | 157 | 2.2 | -0.6 (-18.3, 20.9) | 0.95 |
|  | Male | 0-6 | 94 | 2.6 | 24.2 (-9.8, 71.0) | 0.18 |
|  |  | 7-13 | 94 | 2.2 | 20.0 (-6.3, 53.8) | 0.15 |
|  |  | 14-20 | 93 | 2.2 | 19.3 (-7.3, 53.4) | 0.17 |
| BC | Female | 0-6 | 161 | 0.1 | 43.4 (9.2, 88.2) | 0.01 |
|  |  | 7-13 | 162 | 0.1 | 28.6 (0.6, 64.3) | 0.05 |
|  |  | 14-20 | 162 | 0.1 | -5.0 (-27.8, 25.1) | 0.72 |
|  | Male | 0-6 | 94 | 0.1 | 10.8 (-22.1, 57.4) | 0.57 |
|  |  | 7-13 | 94 | 0.1 | 2.0 (-28.2, 44.8) | 0.91 |
|  |  | 14-20 | 94 | 0.1 | 28.2 (-11.3, 85.3) | 0.19 |
| NO_2_ | Female | 0-6 | 160 | 3.7 | 62.6 (7.1, 147.0) | 0.02 |
|  |  | 7-13 | 160 | 3.7 | 9.8 (-26.9, 64.8) | 0.65 |
|  |  | 14-20 | 161 | 3.8 | -0.1 (-34.7, 52.8) | 1.00 |
|  | Male | 0-6 | 94 | 3.7 | 45.6 (-11.7, 140.1) | 0.14 |
|  |  | 7-13 | 93 | 3.7 | 30.8 (-25.4, 129.3) | 0.35 |
|  |  | 14-20 | 94 | 3.8 | 5.6 (-43.4, 97.3) | 0.86 |
| CO | Female | 0-6 | 156 | 0.1 | 5.1 (-8.4, 20.5) | 0.48 |
|  |  | 7-13 | 158 | 0.1 | 11.5 (-3.5, 28.7) | 0.14 |
|  |  | 14-20 | 155 | 0.1 | 1.8 (-19.3, 28.3) | 0.88 |
|  | Male | 0-6 | 92 | 0.1 | 36.6 (-9.2, 105.4) | 0.13 |
|  |  | 7-13 | 94 | 0.1 | 29.7 (-1.9, 71.4) | 0.07 |
|  |  | 14-20 | 93 | 0.1 | 86.3 (17.8, 194.4) | 0.01 |
| DC | Female | 0-6 | 161 | 0.1 | 16.5 (-8.0, 47.5) | 0.20 |
|  |  | 7-13 | 162 | 0.1 | 5.5 (-15.4, 31.7) | 0.63 |
|  |  | 14-20 | 162 | 0.1 | -22.3 (-41.9, 4.0) | 0.09 |
|  | Male | 0-6 | 94 | 0.1 | 0.3 (-26.8, 37.4) | 0.99 |
|  |  | 7-13 | 94 | 0.1 | -6.7 (-34.0, 31.8) | 0.69 |
|  |  | 14-20 | 94 | 0.1 | -14.5 (-41.4, 24.7) | 0.42 |
| UFP | Female | 0-6 | 145 | 1182.5 | -0.1 (-23.6, 30.6) | 0.99 |
|  |  | 7-13 | 129 | 1170.8 | 15.6 (-11.4, 50.9) | 0.29 |
|  |  | 14-20 | 140 | 1140.5 | 10.3 (-17.4, 47.2) | 0.51 |
|  | Male | 0-6 | 84 | 1182.5 | -4.4 (-35.1, 40.9) | 0.82 |
|  |  | 7-13 | 82 | 1170.8 | -3.4 (-34.5, 42.3) | 0.86 |
|  |  | 14-20 | 80 | 1140.5 | -10.2 (-40.5, 35.6) | 0.61 |
| AMP | Female | 0-6 | 145 | 278.6 | 19.9 (-9.5, 58.9) | 0.21 |
|  |  | 7-13 | 129 | 269.8 | 13.0 (-15.7, 51.5) | 0.41 |
|  |  | 14-20 | 140 | 280.5 | -15.9 (-38.2, 14.3) | 0.27 |
|  | Male | 0-6 | 84 | 278.6 | 4.7 (-30.1, 56.9) | 0.82 |
|  |  | 7-13 | 82 | 269.8 | 13.1 (-22.7, 65.6) | 0.53 |
|  |  | 14-20 | 80 | 280.5 | 14.8 (-21.7, 68.3) | 0.48 |
| SO_2_ | Female | 0-6 | 162 | 0.1 | 11.0 (-15.0, 44.9) | 0.44 |
|  |  | 7-13 | 162 | 0.1 | 6.5 (-19.3, 40.5) | 0.66 |
|  |  | 14-20 | 162 | 0.1 | 18.2 (-11.3, 57.4) | 0.25 |
|  | Male | 0-6 | 94 | 0.1 | 0.5 (-36.9, 60.0) | 0.98 |
|  |  | 7-13 | 94 | 0.1 | 9.2 (-19.2, 47.6) | 0.57 |
|  |  | 14-20 | 94 | 0.1 | -1.2 (-34.4, 48.7) | 0.95 |
| O_3_ | Female | 0-6 | 156 | 0.01 | -12.9 (-51.1, 55.1) | 0.64 |
|  |  | 7-13 | 159 | 0.01 | 9.9 (-35.4, 87.1) | 0.73 |
|  |  | 14-20 | 158 | 0.01 | -24.1 (-56.5, 32.4) | 0.33 |
|  | Male | 0-6 | 94 | 0.01 | -13.5 (-59.5, 84.5) | 0.71 |
|  |  | 7-13 | 91 | 0.01 | -6.1 (-51.3, 81.1) | 0.85 |
|  |  | 14-20 | 90 | 0.01 | 12.0 (-46.3, 133.6) | 0.76 |

**S6 Table:** Excess rate of viral respiratory infection hospital admissions associated with interquartile range increases (IQR) in pollutant concentrations, by lag time and area deprivation index score (<9 and ≥9).

| **Air pollutants** | **Population Characteristics** | **Lag** | **N** | **IQR** | **Excess Rate % (95% CI)** | **p-value** |
| --- | --- | --- | --- | --- | --- | --- |
| PM_2.5_ | ADI<9 | 0-6 | 53 | 2.6 | 27.7 (1.1, 61.3) | 0.04 |
|  |  | 7-13 | 53 | 2.2 | 21.2 (-0.9, 48.3) | 0.06 |
|  |  | 14-20 | 53 | 2.2 | -2.6 (-19.3, 17.6) | 0.78 |
|  | ADI≥9 | 0-6 | 131 | 2.6 | 24.7 (-3.9, 61.8) | 0.10 |
|  |  | 7-13 | 131 | 2.2 | 16.0 (-6.4, 43.7) | 0.17 |
|  |  | 14-20 | 128 | 2.2 | -3.4 (-22.1, 19.6) | 0.75 |
| BC | ADI<9 | 0-6 | 52 | 0.1 | 30.9 (-3.1, 77.0) | 0.08 |
|  |  | 7-13 | 53 | 0.1 | 21.0 (-6.5, 56.7) | 0.15 |
|  |  | 14-20 | 53 | 0.1 | 0.1 (-24.6, 32.8) | 1.00 |
|  | ADI≥9 | 0-6 | 131 | 0.1 | 20.6 (-10.0, 61.7) | 0.21 |
|  |  | 7-13 | 131 | 0.1 | 23.8 (-6.6, 64.0) | 0.14 |
|  |  | 14-20 | 131 | 0.1 | -9.5 (-33.7, 23.6) | 0.53 |
| NO_2_ | ADI<9 | 0-6 | 53 | 3.7 | 64.9 (12.1, 142.5) | 0.01 |
|  |  | 7-13 | 53 | 3.7 | 21.1 (-17.9, 78.6) | 0.33 |
|  |  | 14-20 | 53 | 3.8 | -1.0 (-34.8, 50.4) | 0.96 |
|  | ADI≥9 | 0-6 | 130 | 3.7 | 42.7 (-4.3, 112.6) | 0.08 |
|  |  | 7-13 | 130 | 3.7 | 18.0 (-22.9, 80.7) | 0.45 |
|  |  | 14-20 | 130 | 3.8 | -10.5 (-43.7, 42.3) | 0.64 |
| CO | ADI<9 | 0-6 | 52 | 0.1 | 4.9 (-12.5, 25.6) | 0.61 |
|  |  | 7-13 | 52 | 0.1 | 17.1 (1.3, 35.3) | 0.03 |
|  |  | 14-20 | 51 | 0.1 | 27.3 (-1.1, 63.7) | 0.06 |
|  | ADI≥9 | 0-6 | 127 | 0.1 | 13.1 (-5.5, 35.2) | 0.18 |
|  |  | 7-13 | 128 | 0.1 | 25.4 (1.7, 54.6) | 0.03 |
|  |  | 14-20 | 127 | 0.1 | 6.3 (-15.9, 34.5) | 0.61 |
| DC | ADI<9 | 0-6 | 52 | 0.1 | 12.6 (-11.6, 43.3) | 0.34 |
|  |  | 7-13 | 53 | 0.1 | 1.7 (-19.0, 27.8) | 0.88 |
|  |  | 14-20 | 53 | 0.1 | -21.2 (-40.4, 4.3) | 0.10 |
|  | ADI≥9 | 0-6 | 131 | 0.1 | 10.3 (-14.1, 41.8) | 0.44 |
|  |  | 7-13 | 131 | 0.1 | 0.2 (-23.4, 31.2) | 0.99 |
|  |  | 14-20 | 131 | 0.1 | -26.4 (-46.7, 1.4) | 0.06 |
| UFP | ADI<9 | 0-6 | 47 | 1182.5 | -3.7 (-27.6, 27.9) | 0.79 |
|  |  | 7-13 | 50 | 1170.8 | 8.5 (-17.2, 42.3) | 0.55 |
|  |  | 14-20 | 48 | 1140.5 | 5.4 (-23.7, 45.4) | 0.75 |
|  | ADI≥9 | 0-6 | 117 | 1182.5 | -8.5 (-32.0, 23.1) | 0.56 |
|  |  | 7-13 | 103 | 1170.8 | 21.6 (-11.1, 66.2) | 0.22 |
|  |  | 14-20 | 108 | 1140.5 | -8.2 (-33.6, 26.8) | 0.60 |
| AMP | ADI<9 | 0-6 | 47 | 278.6 | 8.3 (-19.7, 46.0) | 0.60 |
|  |  | 7-13 | 50 | 269.8 | 16.3 (-13.3, 56.1) | 0.31 |
|  |  | 14-20 | 48 | 280.5 | -9.6 (-33.6, 22.9) | 0.52 |
|  | ADI≥9 | 0-6 | 117 | 278.6 | 19.3 (-13.1, 63.7) | 0.28 |
|  |  | 7-13 | 103 | 269.8 | 10.1 (-21.2, 53.7) | 0.57 |
|  |  | 14-20 | 108 | 280.5 | -22.1 (-45.0, 10.4) | 0.16 |
| SO_2_ | ADI<9 | 0-6 | 53 | 0.1 | 15.1 (-12.7, 51.9) | 0.32 |
|  |  | 7-13 | 53 | 0.1 | 19.2 (-6.1, 51.2) | 0.15 |
|  |  | 14-20 | 53 | 0.1 | 11.9 (-15.3, 47.9) | 0.43 |
|  | ADI≥9 | 0-6 | 131 | 0.1 | 15.9 (-17.3, 62.3) | 0.39 |
|  |  | 7-13 | 131 | 0.1 | 16.3 (-11.5, 52.9) | 0.28 |
|  |  | 14-20 | 131 | 0.1 | -3.6 (-31.0, 34.6) | 0.83 |
| O_3_ | ADI<9 | 0-6 | 53 | 0.01 | -33.1 (-62.8, 20.3) | 0.18 |
|  |  | 7-13 | 53 | 0.01 | -13.7 (-49.2, 46.5) | 0.59 |
|  |  | 14-20 | 51 | 0.01 | -13.6 (-51.3, 53.2) | 0.62 |
|  | ADI≥9 | 0-6 | 127 | 0.01 | -21.8 (-57.9, 45.1) | 0.44 |
|  |  | 7-13 | 128 | 0.01 | -3.8 (-45.2, 69.0) | 0.89 |
|  |  | 14-20 | 128 | 0.01 | -29.7 (-62.1, 30.2) | 0.26 |

**S7 Table:** Excess rate of respiratory viral infection related hospital admissions associated with interquartile range increases (IQR) in air pollutant concentration, stratified by distance from central monitor, and lag time

| **Air pollutants** | **Population Characteristics** | **Lag** | **N** | **IQR** | **Excess Rate %**  **(95% CI)** | **p-value** |
| --- | --- | --- | --- | --- | --- | --- |
| PM_2.5_ | >5 miles from monitor | 0-6 | 104 | 2.6 | 34.8 (1.5,79) | 0.04 |
|  |  | 7-13 | 104 | 2.2 | 17.2 (-8.3,50) | 0.21 |
|  |  | 14-20 | 101 | 2.2 | 0.3 (-23.3,31.1) | 0.98 |
|  | ≤5 miles from monitor | 0-6 | 152 | 2.6 | 13 (-11.3,44.1) | 0.32 |
|  |  | 7-13 | 152 | 2.2 | 14.5 (-6.5,40.2) | 0.19 |
|  |  | 14-20 | 149 | 2.2 | 8.2 (-10.3,30.5) | 0.41 |
| BC | >5 miles from monitor | 0-6 | 103 | 0.1 | 62.5 (15.5,128.7) | 0.01 |
|  |  | 7-13 | 104 | 0.1 | 9.5 (-19.2,48.5) | 0.56 |
|  |  | 14-20 | 104 | 0.1 | 0.7 (-29.1,43.1) | 0.97 |
|  | ≤5 miles from monitor | 0-6 | 152 | 0.1 | 12.1 (-15.3,48.2) | 0.42 |
|  |  | 7-13 | 152 | 0.1 | 28.7 (-1.7,68.3) | 0.07 |
|  |  | 14-20 | 152 | 0.1 | 7.3 (-18.8,41.9) | 0.62 |
| NO_2_ | >5 miles from monitor | 0-6 | 103 | 3.7 | 115 (23.3,274.6) | 0.01 |
|  |  | 7-13 | 103 | 3.7 | 19.3 (-30.4,104.5) | 0.52 |
|  |  | 14-20 | 103 | 3.8 | 3.1 (-40.1,77.5) | 0.91 |
|  | ≤5 miles from monitor | 0-6 | 151 | 3.7 | 27 (-15.1,89.9) | 0.24 |
|  |  | 7-13 | 150 | 3.7 | 16.9 (-22.8,77) | 0.46 |
|  |  | 14-20 | 152 | 3.8 | -0.8 (-37.4,57.4) | 0.97 |
| CO | >5 miles from monitor | 0-6 | 101 | 0.1 | 15.4 (-6.3,42) | 0.18 |
|  |  | 7-13 | 102 | 0.1 | 17.4 (-6.3,47.2) | 0.16 |
|  |  | 14-20 | 101 | 0.1 | 5.2 (-18.9,36.6) | 0.7 |
|  | ≤5 miles from monitor | 0-6 | 147 | 0.1 | 2.9 (-13.3,22.2) | 0.74 |
|  |  | 7-13 | 150 | 0.1 | 15.6 (-0.4,34.3) | 0.06 |
|  |  | 14-20 | 147 | 0.1 | 34 (3.3,73.9) | 0.03 |
| DC | >5 miles from monitor | 0-6 | 103 | 0.1 | 35.2 (2.8,77.8) | 0.03 |
|  |  | 7-13 | 104 | 0.1 | -4.9 (-28.9,27.2) | 0.73 |
|  |  | 14-20 | 104 | 0.1 | -36.7 (-56.6,-7.7) | 0.02 |
|  | ≤5 miles from monitor | 0-6 | 152 | 0.1 | -9.4 (-30.8,18.5) | 0.47 |
|  |  | 7-13 | 152 | 0.1 | 8.4 (-15.3,38.6) | 0.52 |
|  |  | 14-20 | 152 | 0.1 | -7.6 (-30.9,23.7) | 0.6 |
| UFP | >5 miles from monitor | 0-6 | 93 | 1182.5 | 7.9 (-25.2,55.6) | 0.69 |
|  |  | 7-13 | 89 | 1170.8 | 10.8 (-21.8,56.8) | 0.56 |
|  |  | 14-20 | 92 | 1140.5 | 25.4 (-12.7,80.2) | 0.22 |
|  | ≤5 miles from monitor | 0-6 | 136 | 1182.5 | -6.6 (-29.2,23.2) | 0.63 |
|  |  | 7-13 | 122 | 1170.8 | 8.3 (-18.5,43.8) | 0.58 |
|  |  | 14-20 | 128 | 1140.5 | -11 (-35.3,22.4) | 0.47 |
| AMP | >5 miles from monitor | 0-6 | 93 | 278.6 | 37.3 (-3.5,95.5) | 0.08 |
|  |  | 7-13 | 89 | 269.8 | 10.4 (-22.9,58.1) | 0.59 |
|  |  | 14-20 | 92 | 280.5 | -11.7 (-39.2,28.1) | 0.51 |
|  | ≤5 miles from monitor | 0-6 | 136 | 278.6 | 0.2 (-26.2,36.3) | 0.99 |
|  |  | 7-13 | 122 | 269.8 | 14.4 (-15.6,55) | 0.39 |
|  |  | 14-20 | 128 | 280.5 | 0.2 (-26.6,36.6) | 0.99 |
| SO_2_ | >5 miles from monitor | 0-6 | 104 | 0.1 | 24.7 (-11.5,75.8) | 0.21 |
|  |  | 7-13 | 104 | 0.1 | 4.2 (-24.7,44) | 0.81 |
|  |  | 14-20 | 104 | 0.1 | 18.4 (-19,73.2) | 0.38 |
|  | ≤5 miles from monitor | 0-6 | 152 | 0.1 | -4.2 (-30.2,31.6) | 0.79 |
|  |  | 7-13 | 152 | 0.1 | 8.4 (-16.6,40.9) | 0.55 |
|  |  | 14-20 | 152 | 0.1 | 7.7 (-20,44.9) | 0.63 |
| O_3_ | >5 miles from monitor | 0-6 | 100 | 0.01 | 15.1 (-43.8,135.7) | 0.7 |
|  |  | 7-13 | 101 | 0.01 | 19.9 (-38.1,132.4) | 0.59 |
|  |  | 14-20 | 101 | 0.01 | 24.6 (-38.2,151) | 0.54 |
|  | ≤5 miles from monitor | 0-6 | 150 | 0.01 | -25.3 (-59,35.9) | 0.34 |
|  |  | 7-13 | 149 | 0.01 | -8 (-46,57) | 0.76 |
|  |  | 14-20 | 147 | 0.01 | -27.7 (-59.6,29.3) | 0.27 |

**S8 Table:** Smoking status specific excess rate of viral infections related hospital admissions associated with interquartile range increases (IQR) in air pollutant concentration, by lag time

| **Air pollutants** | **Population Characteristics** | **Lag** | **N** | **IQR** | **Excess Rate %**  **(95% CI)** | **p-value** |
| --- | --- | --- | --- | --- | --- | --- |
| PM_2.5_ | Active (within 3 months) | 0-6 | 81 | 2.6 | 9.1 (-21.6, 51.9) | 0.60 |
|  |  | 7-13 | 81 | 2.2 | 3.1 (-20.2, 33.1) | 0.82 |
|  |  | 14-20 | 77 | 2.2 | -3.3 (-27.4, 28.7) | 0.82 |
|  | Never | 0-6 | 76 | 2.6 | 7.0 (-22.3, 47.3) | 0.68 |
|  |  | 7-13 | 76 | 2.2 | 10.8 (-17.4, 48.8) | 0.49 |
|  |  | 14-20 | 75 | 2.2 | 15.5 (-12.6, 52.8) | 0.31 |
|  | Past | 0-6 | 99 | 2.6 | 54.9 (13.4, 111.6) | 0.01 |
|  |  | 7-13 | 99 | 2.2 | 32.3 (1.5, 72.5) | 0.04 |
|  |  | 14-20 | 98 | 2.2 | 5.3 (-17.6, 34.7) | 0.68 |
| BC | Active (within 3 months) | 0-6 | 81 | 0.1 | 29.7 (-11.0, 88.8) | 0.18 |
|  |  | 7-13 | 81 | 0.1 | -5.1 (-34.1, 36.6) | 0.78 |
|  |  | 14-20 | 81 | 0.1 | -11.8 (-40.3, 30.3) | 0.53 |
|  | Never | 0-6 | 76 | 0.1 | 24.6 (-18.4, 90.4) | 0.31 |
|  |  | 7-13 | 76 | 0.1 | 50.9 (-1.1, 130.1) | 0.06 |
|  |  | 14-20 | 76 | 0.1 | 25.3 (-19.0, 93.9) | 0.31 |
|  | Past | 0-6 | 98 | 0.1 | 36.8 (-2.0, 91.0) | 0.07 |
|  |  | 7-13 | 99 | 0.1 | 23.6 (-8.4, 66.9) | 0.17 |
|  |  | 14-20 | 99 | 0.1 | 7.8 (-23.9, 52.6) | 0.67 |
| NO_2_ | Active (within 3 months) | 0-6 | 81 | 3.7 | 18.3 (-31.2, 103.3) | 0.54 |
|  |  | 7-13 | 79 | 3.7 | -16.6 (-53.9, 50.9) | 0.55 |
|  |  | 14-20 | 81 | 3.8 | -33.1 (-66.3, 32.9) | 0.25 |
|  | Never | 0-6 | 75 | 3.7 | 24.0 (-31.5, 124.6) | 0.48 |
|  |  | 7-13 | 75 | 3.7 | 72.7 (-9.5, 229.8) | 0.10 |
|  |  | 14-20 | 75 | 3.8 | 32.8 (-26.1, 138.7) | 0.34 |
|  | Past | 0-6 | 98 | 3.7 | 162.4 (47.7, 366.4) | 0.00 |
|  |  | 7-13 | 99 | 3.7 | 20.8 (-27.7, 101.8) | 0.47 |
|  |  | 14-20 | 99 | 3.8 | 4.9 (-40.9, 86.2) | 0.87 |
| CO | Active (within 3 months) | 0-6 | 81 | 0.1 | 6.2 (-17.6, 36.8) | 0.64 |
|  |  | 7-13 | 79 | 0.1 | 11.5 (-8.0, 35.0) | 0.27 |
|  |  | 14-20 | 77 | 0.1 | 9.6 (-25.2, 60.6) | 0.64 |
|  | Never | 0-6 | 73 | 0.1 | 5.7 (-17.1, 34.8) | 0.66 |
|  |  | 7-13 | 75 | 0.1 | 12.6 (-4.5, 32.8) | 0.16 |
|  |  | 14-20 | 75 | 0.1 | 40.8 (-2.2, 102.8) | 0.07 |
|  | Past | 0-6 | 94 | 0.1 | 10.5 (-8.3, 33.2) | 0.29 |
|  |  | 7-13 | 98 | 0.1 | 68.1 (9.6, 157.8) | 0.02 |
|  |  | 14-20 | 96 | 0.1 | 8.0 (-16.1, 39.1) | 0.55 |
| DC | Active (within 3 months) | 0-6 | 81 | 0.1 | 3.9 (-26.2, 46.3) | 0.83 |
|  |  | 7-13 | 81 | 0.1 | -13.1 (-40.9, 27.8) | 0.48 |
|  |  | 14-20 | 81 | 0.1 | -9.7 (-38.7, 32.8) | 0.60 |
|  | Never | 0-6 | 76 | 0.1 | 16.3 (-20.4, 69.9) | 0.44 |
|  |  | 7-13 | 76 | 0.1 | 4.2 (-28.0, 50.7) | 0.83 |
|  |  | 14-20 | 76 | 0.1 | -22.5 (-50.0, 20.3) | 0.26 |
|  | Past | 0-6 | 98 | 0.1 | 10.3 (-16.8, 46.0) | 0.50 |
|  |  | 7-13 | 99 | 0.1 | 8.3 (-16.8, 41.0) | 0.55 |
|  |  | 14-20 | 99 | 0.1 | -27.5 (-50.7, 6.8) | 0.10 |
| UFP | Active (within 3 months) | 0-6 | 71 | 1182.5 | -6.9 (-35.5, 34.5) | 0.70 |
|  |  | 7-13 | 66 | 1170.8 | 15.3 (-19.8, 65.6) | 0.44 |
|  |  | 14-20 | 70 | 1140.5 | 0.0 (-32.6, 48.3) | 1.00 |
|  | Never | 0-6 | 69 | 1182.5 | -12.5 (-43.6, 35.7) | 0.55 |
|  |  | 7-13 | 63 | 1170.8 | 12.9 (-25.5, 70.9) | 0.57 |
|  |  | 14-20 | 68 | 1140.5 | -4.7 (-38.4, 47.5) | 0.83 |
|  | Past | 0-6 | 89 | 1182.5 | 14.8 (-19.7, 64.1) | 0.45 |
|  |  | 7-13 | 82 | 1170.8 | -2.2 (-32.7, 42.2) | 0.91 |
|  |  | 14-20 | 82 | 1140.5 | 14.6 (-23.7, 72.2) | 0.51 |
| AMP | Active (within 3 months) | 0-6 | 71 | 278.6 | 9.3 (-27.3, 64.2) | 0.67 |
|  |  | 7-13 | 66 | 269.8 | -1.9 (-34.9, 47.9) | 0.93 |
|  |  | 14-20 | 70 | 280.5 | -21.0 (-48.7, 21.6) | 0.28 |
|  | Never | 0-6 | 69 | 278.6 | 5.5 (-33.4, 67.1) | 0.82 |
|  |  | 7-13 | 63 | 269.8 | 0.3 (-34.3, 53.2) | 0.99 |
|  |  | 14-20 | 68 | 280.5 | 5.4 (-31.4, 61.9) | 0.81 |
|  | Past | 0-6 | 89 | 278.6 | 32.2 (-8.0, 90.0) | 0.13 |
|  |  | 7-13 | 82 | 269.8 | 34.3 (-8.2, 96.6) | 0.13 |
|  |  | 14-20 | 82 | 280.5 | -4.3 (-35.5, 41.9) | 0.83 |
| SO_2_ | Active (within 3 months) | 0-6 | 81 | 0.1 | -11.0 (-43.3, 39.7) | 0.61 |
|  |  | 7-13 | 81 | 0.1 | 9.2 (-23.1, 55.0) | 0.62 |
|  |  | 14-20 | 81 | 0.1 | 15.3 (-28.7, 86.6) | 0.56 |
|  | Never | 0-6 | 76 | 0.1 | 14.7 (-23.5, 71.8) | 0.51 |
|  |  | 7-13 | 76 | 0.1 | 4.8 (-25.0, 46.5) | 0.78 |
|  |  | 14-20 | 76 | 0.1 | 4.0 (-30.0, 54.4) | 0.85 |
|  | Past | 0-6 | 99 | 0.1 | 20.8 (-17.1, 76.0) | 0.33 |
|  |  | 7-13 | 99 | 0.1 | 4.4 (-28.6, 52.6) | 0.82 |
|  |  | 14-20 | 99 | 0.1 | 12.6 (-22.0, 62.7) | 0.53 |
| O_3_ | Active (within 3 months) | 0-6 | 79 | 0.01 | -21.5 (-66.3, 82.7) | 0.57 |
|  |  | 7-13 | 79 | 0.01 | -23.4 (-63.4, 60.3) | 0.48 |
|  |  | 14-20 | 80 | 0.01 | 17.4 (-46.8, 159.3) | 0.69 |
|  | Never | 0-6 | 73 | 0.01 | -58.0 (-82.8, 2.8) | 0.06 |
|  |  | 7-13 | 75 | 0.01 | 1.3 (-52.8, 117.4) | 0.97 |
|  |  | 14-20 | 75 | 0.01 | -15.1 (-63.6, 98.1) | 0.71 |
|  | Past | 0-6 | 98 | 0.01 | 68.1 (-18.2, 245.2) | 0.16 |
|  |  | 7-13 | 96 | 0.01 | 32.6 (-31.2, 155.7) | 0.40 |
|  |  | 14-20 | 93 | 0.01 | -26.2 (-63.0, 47.5) | 0.39 |

**S9 Table:** Lung disease specific excess rate of viral infections related hospital admissions associated with interquartile range increases (IQR) in air pollutant concentration, by lag time

| **Air pollutants** | **Population Characteristics** | **Lag** | **N** | **IQR** | **Excess Rate % (95% CI)** | **p-value** |
| --- | --- | --- | --- | --- | --- | --- |
| PM_2.5_ | Asthma/COPD | 0-6 | 133 | 2.6 | 34.2 (3.9, 73.4) | 0.02 |
|  |  | 7-13 | 133 | 2.2 | 21.1 (-2.6, 50.6) | 0.09 |
|  |  | 14-20 | 130 | 2.2 | 9.3 (-12.0, 35.8) | 0.42 |
|  | No Asthma/COPD | 0-6 | 88 | 2.6 | 26.4 (-8.2, 73.9) | 0.15 |
|  |  | 7-13 | 88 | 2.2 | 14.2 (-12.6, 49.1) | 0.33 |
|  |  | 14-20 | 86 | 2.2 | 17.8 (-9.5, 53.5) | 0.22 |
| BC | Asthma/COPD | 0-6 | 133 | 0.1 | 48.1 (11.0, 97.7) | 0.01 |
|  |  | 7-13 | 133 | 0.1 | 34.3 (3.3, 74.7) | 0.03 |
|  |  | 14-20 | 133 | 0.1 | 16.4 (-12.8, 55.3) | 0.30 |
|  | No Asthma/COPD | 0-6 | 88 | 0.1 | 13.0 (-23.3, 66.6) | 0.54 |
|  |  | 7-13 | 88 | 0.1 | -4.2 (-34.9, 41.0) | 0.83 |
|  |  | 14-20 | 88 | 0.1 | -2.1 (-34.3, 45.9) | 0.92 |
| NO_2_ | Asthma/COPD | 0-6 | 133 | 3.7 | 78.9 (14.0, 180.6) | 0.01 |
|  |  | 7-13 | 131 | 3.7 | 28.6 (-17.1, 99.3) | 0.26 |
|  |  | 14-20 | 132 | 3.8 | 6.6 (-36.0, 77.5) | 0.81 |
|  | No Asthma/COPD | 0-6 | 87 | 3.7 | 52.7 (-12.7, 167.0) | 0.14 |
|  |  | 7-13 | 87 | 3.7 | 10.5 (-41.8, 109.8) | 0.76 |
|  |  | 14-20 | 88 | 3.8 | 12.5 (-37.6, 102.7) | 0.69 |
| CO | Asthma/COPD | 0-6 | 129 | 0.1 | 14.7 (-4.2, 37.2) | 0.14 |
|  |  | 7-13 | 131 | 0.1 | 18.1 (-4.8, 46.4) | 0.13 |
|  |  | 14-20 | 128 | 0.1 | 20.7 (-11.9, 65.4) | 0.24 |
|  | No Asthma/COPD | 0-6 | 87 | 0.1 | 0.4 (-18.8, 24.2) | 0.97 |
|  |  | 7-13 | 87 | 0.1 | 12.5 (-5.6, 34.2) | 0.19 |
|  |  | 14-20 | 85 | 0.1 | 20.3 (-3.3, 49.7) | 0.10 |
| DC | Asthma/COPD | 0-6 | 133 | 0.1 | 17.4 (-7.3, 48.8) | 0.18 |
|  |  | 7-13 | 133 | 0.1 | 10.6 (-12.9, 40.3) | 0.41 |
|  |  | 14-20 | 133 | 0.1 | -8.6 (-32.1, 23.2) | 0.56 |
|  | No Asthma/COPD | 0-6 | 88 | 0.1 | 10.0 (-23.3, 57.7) | 0.60 |
|  |  | 7-13 | 88 | 0.1 | -1.5 (-31.3, 41.1) | 0.93 |
|  |  | 14-20 | 88 | 0.1 | -36.4 (-59.9, 0.9) | 0.05 |
| UFP | Asthma/COPD | 0-6 | 120 | 1182.5 | 7.4 (-20.1, 44.2) | 0.64 |
|  |  | 7-13 | 108 | 1170.8 | 13.9 (-16.0, 54.5) | 0.40 |
|  |  | 14-20 | 114 | 1140.5 | -5.9 (-32.0, 30.2) | 0.71 |
|  | No Asthma/COPD | 0-6 | 80 | 1182.5 | -8.4 (-38.8, 37.1) | 0.67 |
|  |  | 7-13 | 76 | 1170.8 | 13.2 (-22.9, 66.1) | 0.53 |
|  |  | 14-20 | 78 | 1140.5 | 24.4 (-16.7, 85.9) | 0.29 |
| AMP | Asthma/COPD | 0-6 | 120 | 278.6 | 32.6 (-3.1, 81.5) | 0.08 |
|  |  | 7-13 | 108 | 269.8 | 15.5 (-15.8, 58.4) | 0.37 |
|  |  | 14-20 | 114 | 280.5 | -18.0 (-41.6, 15.0) | 0.25 |
|  | No Asthma/COPD | 0-6 | 80 | 278.6 | 2.7 (-31.2, 53.2) | 0.90 |
|  |  | 7-13 | 76 | 269.8 | 21.5 (-18.9, 82.0) | 0.35 |
|  |  | 14-20 | 78 | 280.5 | 23.6 (-16.0, 82.0) | 0.28 |
| SO_2_ | Asthma/COPD | 0-6 | 133 | 0.1 | 3.1 (-24.7, 41.2) | 0.85 |
|  |  | 7-13 | 133 | 0.1 | 9.1 (-16.9, 43.2) | 0.53 |
|  |  | 14-20 | 133 | 0.1 | -4.6 (-33.4, 36.6) | 0.80 |
|  | No Asthma/COPD | 0-6 | 88 | 0.1 | 27.2 (-15.6, 91.8) | 0.25 |
|  |  | 7-13 | 88 | 0.1 | 7.0 (-25.7, 54.2) | 0.72 |
|  |  | 14-20 | 88 | 0.1 | 38.6 (-2.5, 97.1) | 0.07 |
| O_3_ | Asthma/COPD | 0-6 | 129 | 0.01 | -35.4 (-65.9, 22.6) | 0.18 |
|  |  | 7-13 | 129 | 0.01 | -3.8 (-45.8, 70.8) | 0.90 |
|  |  | 14-20 | 130 | 0.01 | -40.8 (-68.2, 10.1) | 0.10 |
|  | No Asthma/COPD | 0-6 | 86 | 0.01 | 20.0 (-47.8, 176.0) | 0.67 |
|  |  | 7-13 | 86 | 0.01 | -5.8 (-53.8, 92.1) | 0.87 |
|  |  | 14-20 | 84 | 0.01 | 66.8 (-22.1, 257.0) | 0.19 |
